# Assessment of dietary supplementation with galactomannan oligosaccharides and phytogenics on gut microbiota of European sea bass (*Dicentrarchus Labrax*) fed low fishmeal and fish oil based diet

**DOI:** 10.1371/journal.pone.0231494

**Published:** 2020-04-16

**Authors:** Simona Rimoldi, Silvia Torrecillas, Daniel Montero, Elisabetta Gini, Alex Makol, Victoria Valdenegro V., Marisol Izquierdo, Genciana Terova

**Affiliations:** 1 Department of Biotechnology and Life Sciences, University of Insubria, Varese, Italy; 2 Grupo de Investigación en Acuicultura (GIA), IU-ECOAQUA, Universidad de Las Palmas de Gran Canaria, Telde, Las Palmas, Canary Islands, Spain; 3 Delacon Biotechnik GmbH, Steyregg, Austria; 4 Biomar A/S. BioMar AS, Trondheim, Norway; University of Illinois, UNITED STATES

## Abstract

There is an increasing interest from the aquafeed industry in functional feeds containing selected additives that improve fish growth performance and health status. Functional feed additives include probiotics, prebiotics, organic acids, and phytogenics (substances derived from plants and their extracts). This study evaluated the effects of dietary inclusion of a mucilage extract rich in galactomannan oligosaccharides (GMOS), a mixture of garlic and labiatae-plants oils (PHYTO), and a combination of them (GMOSPHYTO), on gut microbiota composition of European sea bass (*Dicentrarchus labrax*) fed with a low fishmeal (FM) and fish oil (FO) diet. Three experimental diets and a control diet (plant-based formulation with 10% FM and 6% FO) were tested in a 63-days feeding trial. To analyze the microbiota associated to feeds and the intestinal autochthonous (mucosa-adhered) and allochthonous (transient) microbial communities, the Illumina MiSeq platform for sequencing of 16S rRNA gene and QIIME2 pipeline were used. Metabarcoding analysis of feed-associated bacteria showed that the microbial communities of control (CTRL) feed deeply differed from those of experimental diets. The number of reads was significantly lower in CTRL feed than in other feeds. The OTU (operational taxonomic unit) number was instead similar between the feeds, ranging from 42 to 50 OTUs. The variation of resident gut microbiota induced by diet was lower than the variation of transient intestinal microbiota, because feedstuffs are a major source of allochthonous bacteria, which can temporarily integrate into the gut transient microbiome. However, the composition of transient bacterial communities was not simply a mirror of feed-borne bacteria. Indeed, the microbial profile of feeds was different from both faecal and mucosa profiles. Our findings suggest that the dietary inclusion of GMOS (0.5%) and PHYTO (0.02%) in a low FM and FO diet induces changes in gut microbiota composition of European sea bass. However, if on allochthonous microbiota the combined inclusion of GMOS and PHYTO showed an antagonistic effect on bactericidal activity against Vibrionales, at mucosa level, only GMOSPHYTO diet increased the relative abundance of Bacteroidales, Lactobacillales, and Clostridiales resident bacterial orders. The main beneficial effects of GMOS and PHYTO on gut microbiota are the reduction of coliforms and Vibrionales bacteria, which include several potentially pathogenic species for fish, and the enrichment of gut microbiota composition with butyrate producer taxa. Therefore, these functional ingredients have a great potential to be used as health-promoting agents in the farming of European sea bass and other marine fish.

## Introduction

Farming of carnivorous fish species still relies on fishmeal (FM) and fish oil (FO) that represent the optimal sources of protein and lipids in aquafeeds. However, this dependence has to be overcome because FM and FO are no longer cost-effective and environmentally sustainable resources. Therefore, in the last decades, fish farmers and commercial feed producers have made substantial efforts to progressively reduce the proportion of these products in aquaculture feed, by replacing ground-up forage fish with terrestrial plant-derived protein and lipid sources [1, 2].

However, only a partial replacement of FM and FO is possible without negatively affecting fish growth performance and health status. The main drawbacks of using high levels of plant proteins in carnivorous fish feed are related to several nutritional imbalances and to the presence of a wide variety of anti-nutritional factors that challenge fish intestine functionality and health, thus reducing nutrient absorption and animal growth [3, 4]. Indeed, various studies have described a range of inflammatory gut reactions in carnivorous fish species, such as Atlantic salmon (*Salmo salar*), and European sea bass (*Dicentrarchus labrax*) in response to high replacement levels of marine-derived raw materials with plant-derived raw materials in the diet [4, 5, 6]. The shortening of the primary and secondary intestinal mucosal folds, higher level of infiltrated leucocytes in the *lamina propria* and submucosa, damaged microvilli, altered gut microbiota profiles, and increased gut permeability are the most typical signs of such inflammatory response [5, 6, 7]. These negative-side effects reduce the capacity of enterocytes to absorb nutrients and can promote the translocation of indigenous or opportunistic bacteria, potentially pathogenic for the host [8, 7]. In this context, there is an increasing interest in functional feeds containing selected additives that can help to prevent or mitigate the intestinal epithelium damage caused by extreme plant-based diet formulations [9–13]. Feed additives have quite diverse chemical natures and characteristics; they may be both, nutritive and non-nutritive ingredients and may interact with fish physiology by either direct or indirect mechanisms [14]. Functional feed additives include probiotics, prebiotics, immune-stimulants, organic acids, nucleotides, exogenous enzymes, and phytogenics (substances derived from herbs, spices, other plants and their extracts). Their application into diet formulations targets a specific purpose: probiotics, prebiotics, phytogenics, and immune-stimulants target the improvement of intestinal health, stress, and disease resistance, whereas acidifiers, exogenous enzymes and indirectly probiotics are used to improve fish performance by enhancing feed digestibility, or counteracting the negative effects of antinutrients [14].

Among prebiotics, galactomannans from different sources, have been related to enhanced fish growth performance and health status [15–20]. Galactomannans are heteropolysaccharides structurally composed of D-mannose, which makes up the backbone, and D-galactose that forms single branches along the mannan chain. Galactomannans originate from two sources; the first and main source is represented by plants, in particular, the endosperm of *Leguminosae* family's dicotyledonous plants seeds; the second is represented by microorganisms, such as yeast and fungi, in which galactomannans constitute an essential component of the cell walls.

Galactomannans from plants are in the form of soluble dietary fibers, thereby acting as prebiotics. The basic criteria to consider an ingredient as a prebiotic have been firstly established by Gibson and Roberfroid [21]. They defined a prebiotic as a nondigestible food ingredient that beneficially affects the host by selectively stimulating the growth and/or activity of one or a limited number of bacterial species already resident in the intestine.

Unlike yeasts’ mannans that have been widely investigated in several fish species leading to contrasting results on their effects [22–24, 9, 25–29], galactomannans of plant origin are still scarcely explored as functional ingredients in aquafeeds. Nevertheless, it is known that the improvement of growth performance, health, and disease resistance in fish fed with prebiotics are frequently connected with changes in their gut microbial communities [18, 30–32].

In addition to changes in gut microbiota, other indirect mechanisms of action of prebiotics include bacterial end-products of fermentation. By definition, prebiotics are not digested by the host itself, but are fermented by bacteria present in the host’s gut. The end products of prebiotic fermentation are short chain fatty acids, predominantly acetic, propionic and butyric acids. Among them butyrate is considered to be a preferred energy source for colonocytes and in vertebrates, including fish, plays a pivotal role in the maintenance of overall gut health, intestinal morphology, and function [33–34, 11, 31].

By comparison with prebiotics, only limited information is available on the potential benefits of phytogenics on fish health and, in general, on their application in animal nutrition [35, 13]. These products have great potential to be used in aquaculture due to a number of beneficial biological activities that include growth promotion, appetite stimulation, modulation of immune and antioxidant response in addition to antiparasitic, antibacterial, anaesthetic, and antistress activities [36–43]. These activities are positively reflected on feed palatability, fish digestive functions, and intestinal microbiota structure.

Phytogenic feed additives using plant extracts show greater modes of action in animal nutrition compared to synthetic, nature-identical substances [13, 38]. This advantage is based on the synergistic effects of all agents within a plant, which have not been reduced to the effects of a single lead substance. The challenge is finding the right combinations of natural substances while fully exploiting the synergy among their active ingredients.

Essential oils (EOs) represent the major group of phytogenic feed additives. EOs are a natural mixture of various organic substances synthesized by aromatic plants during secondary metabolism. EOs usually contain high amounts of different compounds, such as terpenes, alcohols, acetones, phenols, acids, aldehydes, and esters [44]. EOs are potent antimicrobial agents against different strains of pathogenic and food borne bacteria [45, 43]. Therefore, due to their antibacterial activity, phytochemicals can exert a prebiotic-like effect modulating fish gut bacterial composition [46, 12].

Accordingly, based on the aforementioned properties of galactomannans and phytogenics, the aim of this study was to investigate the effects of dietary inclusion of galactomannan oligosaccharides (GMOS), a mixture of garlic and labiatae-plants oils (PHYTO), and a combination of them (GMOSPHYTO), on gut microbiota composition of European sea bass fed with a plant-based diet. A 16S metabarcoding approach” was used to characterize gut bacterial communities of European sea bass.

## Material and methods

### Ethics statement

All procedures involving fish complied with the guidelines of the European Union Council (86/609/EU) and Spanish legislation (RD 53/2013) and were approved by Bioethical Committee of the University of Las Palmas de Gran Canaria (Ref. 007/2012 CEBA ULPG).

### Feeding trial, diets, and sampling

Details of the feeding trial, set at Parque Científico-Tecnológico Marino (PCTM) of the University of Las Palmas de Gran Canaria (Telde, Canary Island, Spain), have been described by Torrecillas et al. [20]. Briefly, four isoenergetic and isonitrogenous experimental diets were manufactured by an extrusion process in the BioMar Tech-Centre (Brande, Denmark) starting from a plant-based formulation (10% FM, 6% FO) and containing different additives: 0.5% of galactomannan oligosaccharides from mucilage (GMOS; Delacon, Austria), 0.02% of a mixture of garlic and labiate-plants oils (PHYTO; Delacon, Austria) and a combination of both additives (GMOSPHYTO, 0.52%). Principal ingredients, and proximate composition of diets is reported in Table 1, modified from Torrecillas et al. [20]. GMOS was included in the diet in the mix pre-extrusion process, PHYTO was included in post extrusion process by vacuum coating and homogenized with the dietary fish oil. The GMOS and PHYTO dosages were included in the diets accordingly to commercial recommendations. For GMOS, these recommendations were based on internal trials and other previous studies with similar products for the same fish species. For the blend of herbal extracts, the dietary concentration tested was also based on commercial recommendations, which mainly depended on MIC internal assays against *Vibrio sp*. The stability of the supplements was evaluated before and after feed production, as well as at the beginning of the feeding trial.

**Table 1:** Main ingredients and proximate composition of the diets (modified from Torrecillas et al., 2019).

	Diets (%)			
Ingredients	CONTROL	GMOS	PHYTO	GMOSPHYTO
Fish meal^1^	10	10	10	10
Soya protein concentrate	18.9	18.9	18.9	18.9
Soya meal	12.0	12.0	12.0	12.0
Corn gluten meal	25.0	25.0	25.0	25.0
Wheat	8.7	8.2	8.7	8.2
Wheat gluten	2.0	2.0	2.0	2.0
Guar meal	8.0	8.0	8.0	8.0
Rapeseed extracted	3.0	3.0	3.0	3.0
Fish oil^2^	6.7	6.7	6.7	6.7
Rapeseed oil^3^	5.4	5.4	5.4	5.4
Vitamin and mineral premix^4^	3.7	3.7	3.7	3.7
Antioxidant ^5^	0.06	0.06	0.06	0.06
Galactomannan oligosaccharides^6^	0	0.5	0	0.5
Phytogenic^7^	0	0	0.02	0.02
**Proximate composition (% of dry matter)**				
Crude lipids	19.91	20.44	20.47	20.72
Crude protein	49.30	49.27	49.76	49.85
Moisture	5.10	5.01	5.06	5.17
Ash	7.02	6.41	6.49	6.39
Gross Energy (MJ/kg, as is)	22.07	22.11	22.17	22.25

^1^ South-American, Superprime 68%.

^2^ South American fish oil.

^3^ DLG AS, Denmark.

^4^ Vilomix, Denmark.

^5^ BAROX BECP, Ethoxyquin.

^6^Delacon Biotechnik GmbH, Austria.

^7^Delacon Biotechnik GmbH, Austria.

Nine hundred European sea bass juveniles were randomly distributed in 12 fiberglass tanks of 500 L in an open water system and fed *ad libitum* with four different diets in triplicate for 63 days. At the end of the trial, two fish per replicate (6 fish/diet) were sampled and the whole intestine was aseptically removed. The animals used for sampling were sacrificed by an overdose of anaesthetic (clove oil) using water bath immersion, and all efforts were made to minimize pain, stress, and discomfort in the animals. The faecal matter containing allochthonous (transient) intestinal bacteria was removed from each intestine by squeezing and collected in a sterile tube with 800 μl of Xpedition Lysis/Stabilization Solution (Zymo Research). The autochthonous (adhered) microbiota was obtained by scraping the whole intestinal mucosa (excluding pyloric caeca) with a sterile cotton swab. The tip of swap was immediately immersed in 200 μl of Xpedition Lysis/Stabilization Solution and vortexed to facilitate bacteria releasing as described in Rimoldi et al. [47]. Both faecal and mucosa samples were stored at room temperature for up to 48 hrs until bacterial DNA extraction.

The remained fish following this research were housed at the Las Palmas University facilities to be used in practical lessons for undergraduate students of veterinary and marine sciences faculties, focused to the learning of aquaculture husbandry practices. Fish were not used in further research, due to the influence of experimental diets in the intestine of different groups, giving an erroneous starting point for other feeding trials. Besides, animal’s final weight was high for new trials.

### DNA extraction

The bacterial DNA was extracted from 4 samples from each feed, 6 samples of faeces and 6 of intestinal mucosa per each dietary fish group. For each extraction, 200 mg of feed, 200 mg of faeces, and 200 microliters of mucosal bacteria suspension were used. The extraction of bacterial DNA from the feeds was done in parallel to biological samples, right after the feeding trail.

DNeasy PowerSoil Kit (Qiagen, Italy) was used for extraction, following the manufacturer’s instructions with few modifications at the lysis step. Specifically, lysis was performed in PowerBead Tubes by means of a TissueLyser II (Qiagen, Italy) set at 25 Hz for 2 min. As a negative control for the extraction procedure, a sample with only lysis buffer was processed in parallel with all samples. The concentration of extracted DNA was assessed using a NanoDropTM 2000 Spectrophotometer (Thermo Scientific, Italy). DNA was stored at– 20°C until the PCR reaction was performed.

### 16S Illumina library construction and high-throughput sequencing

Methodology applied for 16S rRNA gene library preparation and sequencing have been described in Rimoldi et al. [47] and Terova et al. [48]. The Illumina protocol “16S Metagenomic Sequencing Library Preparation for Illumina MiSeq System” (#15044223 rev. B) was applied for library preparation. The V3-V4 region was amplified from 50 ng of microbial genomic DNA using Platinum® Taq DNA Polymerase High Fidelity kit (Thermo Fisher Scientific, Italy) and tailed using forward and reverse primers Pro341F (5′-CCTACGGGNBGCASCAG -3′) and Pro805R (5′-GACTACNVGGGTATCTAATCC -3′) selected by Takahashi et al. [49]. The amplicon length was checked on Agilent 2100 Bioanalyzer trace and the expected size was ~550 bp.

Illumina paired-end adapters with unique Nextera XT indexes were ligated to 16S amplicons using Nextera XT Index Kit (Illumina, San Diego, CA). Libraries were purified and normalized using the SequalPrep ™ Normalization Plate kit (Thermo Fisher), pooled at equimolar concentrations and diluted at 6 pM prior to sequencing on the Illumina MiSeq platform (Illumina). Libraries were sequenced with v3 chemistry on 300PE MiSeq runs.

## 16S amplicon sequencing data analysis

Raw FASTQ sequencing data were processed using the open-source bioinformatics pipeline QIIME 2 (v. 2018.4) at the default setting [50]. To reconstruct the original amplicons, the reads generated by three different runs (one with all the samples, and two extra runs with mucosal and faecal samples that gave suboptimal Good’s coverage value (< 99.5%) and low number of reads in the first run) were separately processed with DADA2 [51]. R1 and R2 paired reads were trimmed at both 3’ and 5’ ends using Cutadapt v.2018.4.0 software, filtered for base quality (Q>30) and merged. The remaining high-quality reads were de-replicated to obtain unique sequence (uniques) and chimeric sequences removed using QIIME DADA2 denoise-paired command. Denoised sequences with 99% or higher identity against uniques were de novo clustered into Operational Taxonomic Units (OTUs). Only the OTUs that represented at least 0.005% of total reads were kept. Taxonomy was assigned, down to genus level, using FeatureData [Sequence] artefact against the reference database GreenGenes v.13-8. Reads of mitochondrial or eukaryotic origin were excluded.

Alpha and beta diversity statistics were performed by alpha-phylogenetic and beta-phylogenetic command, respectively. To evaluate the compositional alpha- and beta-diversity, the samples were divided into two macro-groups (faeces+feeds, and mucosae+feeds) and separately analysed. Alpha diversity was calculated based on rarefied OTU table (rarefied at the lowest sample size) using observed OTUs, Shannon, Pielou’s evenness, and Faith PD. Difference in taxonomic profiles among samples (beta diversity) was calculated using weighted (presence/absence/abundance matrix) and unweighted (presence/absence matrix) UniFrac distances [52–53]. The dissimilarity matrices were graphically represented by three-dimensional PCoA plots.

### Statistics

All data were verified for normality and homoscedasticity of variance by Shapiro-Wilk’s and Levene’s test, respectively. Depending on whether the assumptions were satisfied or not, differences between groups were analysed by one-way ANOVA with Tukey’s pairwise test or by nonparametric Kruskal-Wallis followed by Dunn’s post hoc test. Statistical significance was set at p<0.05. All analyses were performed using Past3 software [54].

The number of reads across samples was normalized by sample size and the relative abundance (%) of each taxon was calculated. OTUs assigned to the phylum Cyanobacteria (class Chloroplast) and to mitochondria, were considered potential plant contaminants and were removed from the analysis. For each dietary group, only taxa present in at least half samples and with a mean overall abundance of more than 1% (up to order level) and 0.5% at family and genus level were considered for statistical analysis. Less abundant taxa were indicated as “Others” in the histograms of microbial community profiles of fish. They were not considered for statistical analysis, since we decided to analyse the effects of diet only on the most representative OTUs.

Before being statistically analysed, the resulting microbial profiles relative abundances of each OTU were calculated as the angular transformation (arcsine of the square root). The angular transformation of data is commonly used to make normal a binomial distribution like in our case in which the data were expressed as decimal fractions and percentages.

Multivariate analysis of beta diversity was tested using non-parametric analysis of similarities (ANOSIM) and Adonis tests with 999 permutations.

## Results

### Fish growth parameters and biometry

After 63 days of feeding, the experimental diets did not induce differences in fish growth or diet utilization. All growth performance data concerning the present feeding trial have been recently reported by Torrecillas et al. [20].

### Illumina MiSeq sequencing efficiency

The obtained raw sequencing data were divided into two macro-groups (i.e. faeces+feeds and mucosa+feeds) that were analysed separately, i.e each macro-group indicates the set of samples statistically analysed together by the QIIME2 pipeline. Feeds were sequenced only one time, but they were statistically analysed twice, once per each macro-group.

We obtained a total number of quality-filtered reads of 1,338,727, which corresponded to 34,326 ± 17,538 reads per sample, for faeces+feeds macro-group and 1,114,122 reads, corresponding to 29,319 ± 18,581 reads per sample, for mucosa+feeds macro-group.

An overall of 125 and 210 unique sequences were found in faeces+feeds and mucosa+feeds macro-groups, respectively (S1 Data File). Only two samples (one of GMOS feed and one of mucosa from GMOSPHYTO group) were excluded after rarefaction analysis, since their rarefaction curve did not reach the plateau. On the basis of rarefaction analysis results, faeces+feeds and mucosae+feeds samples were rarefied at 4500 and 5000 reads, respectively. Rarefaction curves of Observed OTUs numbers of two macro-groups of samples have been reported in S1 and S2 Figs. All fastq sequencing files were deposited in the European Nucleotide Archive (EBI ENA) public database under the accession project code: **PRJEB32279**.

### Microbial profile of experimental feeds

Metabarcoding analysis of feed-associated bacteria showed that the microbial communities of CTRL feed deeply differed from those of experimental functional diets GMOSPHYTO, PHYTO, and GMOS. The number of reads taxonomically classified according to Greengenes database was 6,315 ± 760; 30,613 ± 5,521; 42,689 ± 8,558; and 30,953 ± 11,762 for CTRL, GMOS, GMOSPHYTO, and PHYTO diets, respectively. This number was significantly lower in CTRL feed (p<0.05) in comparison to other feed samples. In addition, Pielou’s evenness and Shannon alpha diversity indices of CTRL feed differed from those of functional feeds, being both indices significantly higher in CTRL samples. The OTU number was instead comparable among the diets, ranging from 42 to 50 OTUs (S1 Table).

**Fig 1:**
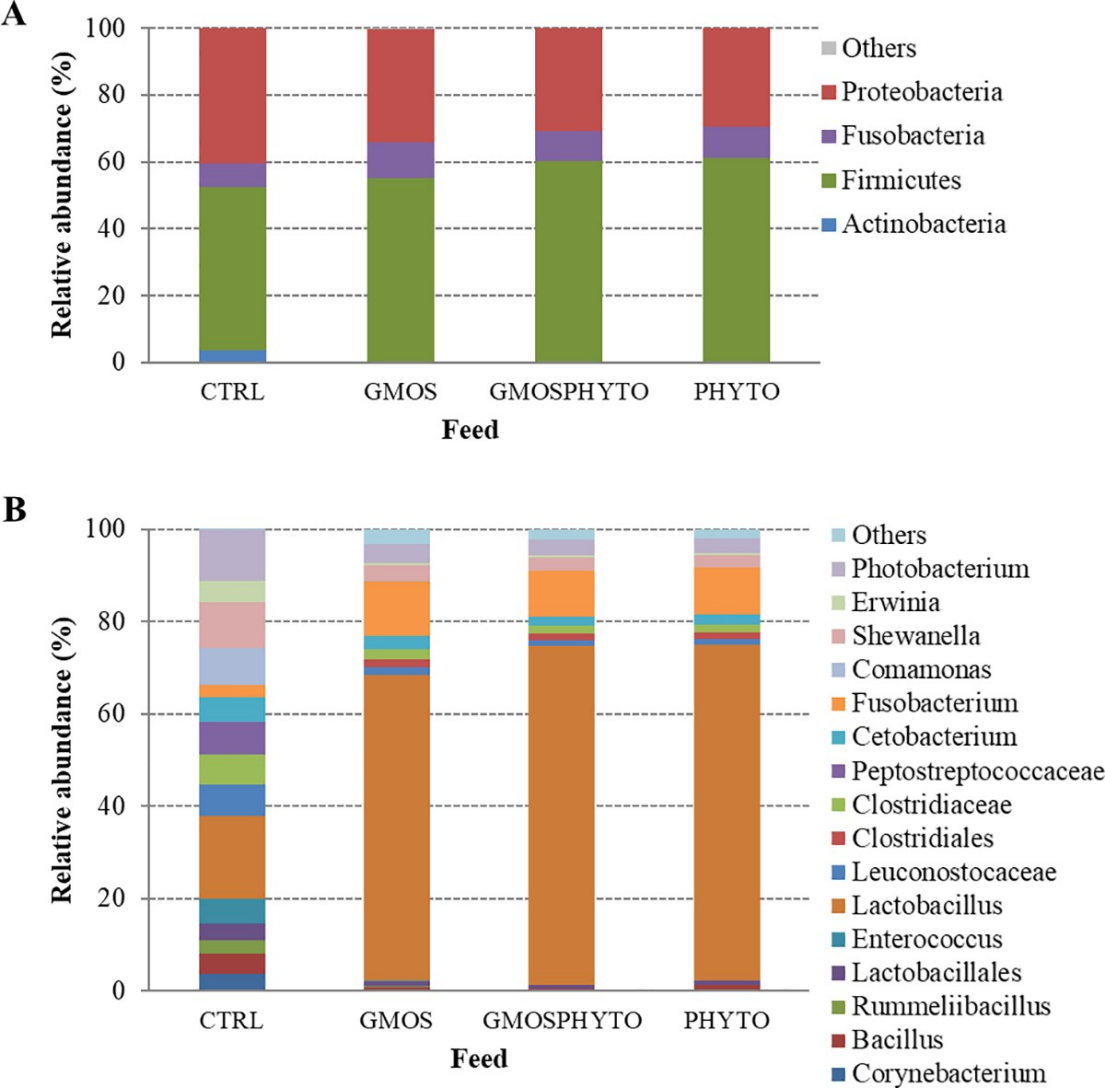
Relative abundance (%) of the most prevalent bacteria in CTRL, GMOS, PHYTO, and GMOSPHYTO feeds at phylum (A), and genus (B) taxonomic level. Only bacteria with an overall abundance of ≥1% were reported. Bacteria with lower abundance were pooled and indicated as “Others”.

After removing the OTUs assigned to eukaryotic sequences, the most abundant bacterial taxa were mainly comprised of 4 phyla, 7 classes, 10 orders, 14 families, and 11 genera. We have presented the profiles of microbial communities for each feed at phylum (Fig 1A) and genus (Fig 1B) taxonomic level and reported the relative abundance (%) of the most abundant taxa found in feed samples (S2 Table).

At phylum level, functional feeds GMOS, GMOSPHYTO, and PHYTO were characterized by higher percentage of Firmicutes (55–61%) than CTRL feed (49%). Conversely, microbiota associated to CTRL feed showed a higher relative abundance of Proteobacteria (40%), mainly represented by the Gammaproteobacteria class, as compared to other feeds (29–34%) (Fig 1A, S2 Table). Accordingly, a high amount of the *Shewanellaceae* (9.9%), *Enterobacteriaceae* (4.6%), and *Vibrionaceae* (11%) families was found in the CTRL diet. Feeds containing prebiotic and/or phytogenic were instead enriched in *Lactobacillaceae* (66–73%).

At genus level, CTRL feed had higher relative abundance of *Corynebacterium* (3.8%), *Enterococcus* (5.5%), *Cetobacterium* (5.24%), *Shewanella* (9.9), *Erwinia* (4.6%), and *Photobacterium* (11.2%), whereas the *Lactobacillus* genus was dominant, with percentages ranging between 66 and 73%, in GMOS, GMOSPHYTO, and PHYTO diets (Fig 1B, S2 Table).

The permutational multivariate analysis made with Adonis and ANOSIM test on both, unweighted and weighted UniFrac distance data, statistically confirmed the differences between feed-associated microbial communities (beta-diversity). Adonis and ANOSIM tests revealed a significant difference (p<0.05) between CTRL and functional feeds (GMOS, GMOSPHYTO, and PHYTO) in both, type (R^2^>0.51, R>0.50) and abundance (R^2^>0.79, R = 1) of taxa found. The results of multivariate analysis are summarized in Table 2.

**Table 2:** Results of permutational multivariate analysis of variance (Adonis) and analysis of similarity (ANOSIM) based on unweighted and weighted UniFrac distance matrices, using abundance data of feed-associated bacterial communities.

Adonis	Unweighted		Weighted	
	***p*-value**	**R2**	***p*-value**	**R2**
diet CTRL vs diet GMOS	**0.022**	0.59	**0.033**	0.79
diet CTRL vs diet GMOSPHYTO	**0.028**	0.67	**0.035**	0.86
diet CTRL vs diet PHYTO	**0.034**	0.51	**0.033**	0.85
diet GMOS vs diet GMOSPHYTO	0.146	0.30	0.421	0.16
diet GMOS vs diet PHYTO	0.500	0.16	0.288	0.19
diet GMOSPHYTO vs diet PHYTO	0.126	0.25	0.624	0.08
**ANOSIM**				
	***p*-value**	**R**	***p*-value**	**R**
diet CTRL vs diet GMOS	**0.036**	0.50	**0.032**	1.00
diet CTRL vs diet GMOSPHYTO	**0.028**	0.62	**0.032**	1.00
diet CTRL vs diet PHYTO	**0.033**	0.59	**0.030**	1.00
diet GMOS vs diet GMOSPHYTO	0.062	0.40	0.176	0.16
diet GMOS vs diet PHYTO	0.709	-0.13	0.167	0.24
diet GMOSPHYTO vs diet PHYTO	0.090	0.16	0.674	-0.11

Significant p-values are presented in bold.

### Microbial profile and dietary modulation of allochthonous gut communities

The whole microbial community profile of 24 faecal samples was mainly composed of 11 phyla, 15 classes, 27 orders, 52 families, and 59 genera. By considering only the most representative taxa, the overall allochthonous microbiota consisted of 6 phyla (Actinobacteria, Bacteriodetes, Firmicutes, Proteobacteria, Spirochaetes, and Tenericutes), 9 classes (Actinobacteria, Bacteroidia, Bacilli, Clostridia, Alphaproteobacteria, Betaproteobacteria, Gammaproteobacteria, [Brevinematae], and Mollicutes), 17 orders (Actinomycetales, Bacteroidales, Bacillales, Lactobacillales, Clostridiales, Rhizobiales, Rickettsiales, Burkholderiales, Neisseriales, Alteromonadales, Enterobacteriales, Oceanospirillales, Pasteurellales, Pseudomonadales, Vibrionales, [Brevimatales], and Mycoplasmatales), 23 families (*Corynebacteriaceae*, *Propionibacteriaceae*, *Bacteroidaceae*, *Bacillaceae*, *Staphylococcaceae*, *Lactobacillaceae*, *Streptococcaceae*, *Lachnospiraceae*, *Ruminococcaceae*, *Aurantimonadaceae*, *Methylobacteriaceae*, *Rhodobacteraceae*, *Comamonadaceae*, *Oxalobacteraceae*, *Neisseriaceae*, *Shewanellaceae*, *Enterobacteriaceae*, *Halomonadaceae*, *Pasteurellaceae*, *Moraxellaceae*, *Vibrionaceae*, *Brevinemataceae*, and *Mycoplasmataceae*), and 24 genera (*Corynebacterium*, *Propionibacterium*, *Bacteroides*, *Staphylococcus*, *Lactobacillus*, *Streptococcus*, *Faecalibacterium*, *Ruminococcus*, *Methylobacterium*, *Paracoccus*, *Aquabacterium*, *Janthinobacterium*, *Glaciecola*, *Shewanella*, *Escherichia*, *Cobetia*, *Aggregatibacter*, *Acinetobacter*, *Enhydrobacter*, *Psychrobacter*, *Enterovibrio*, *Photobacterium*, *Vibrio*, and *Mycoplasma*).

**Fig 2:**
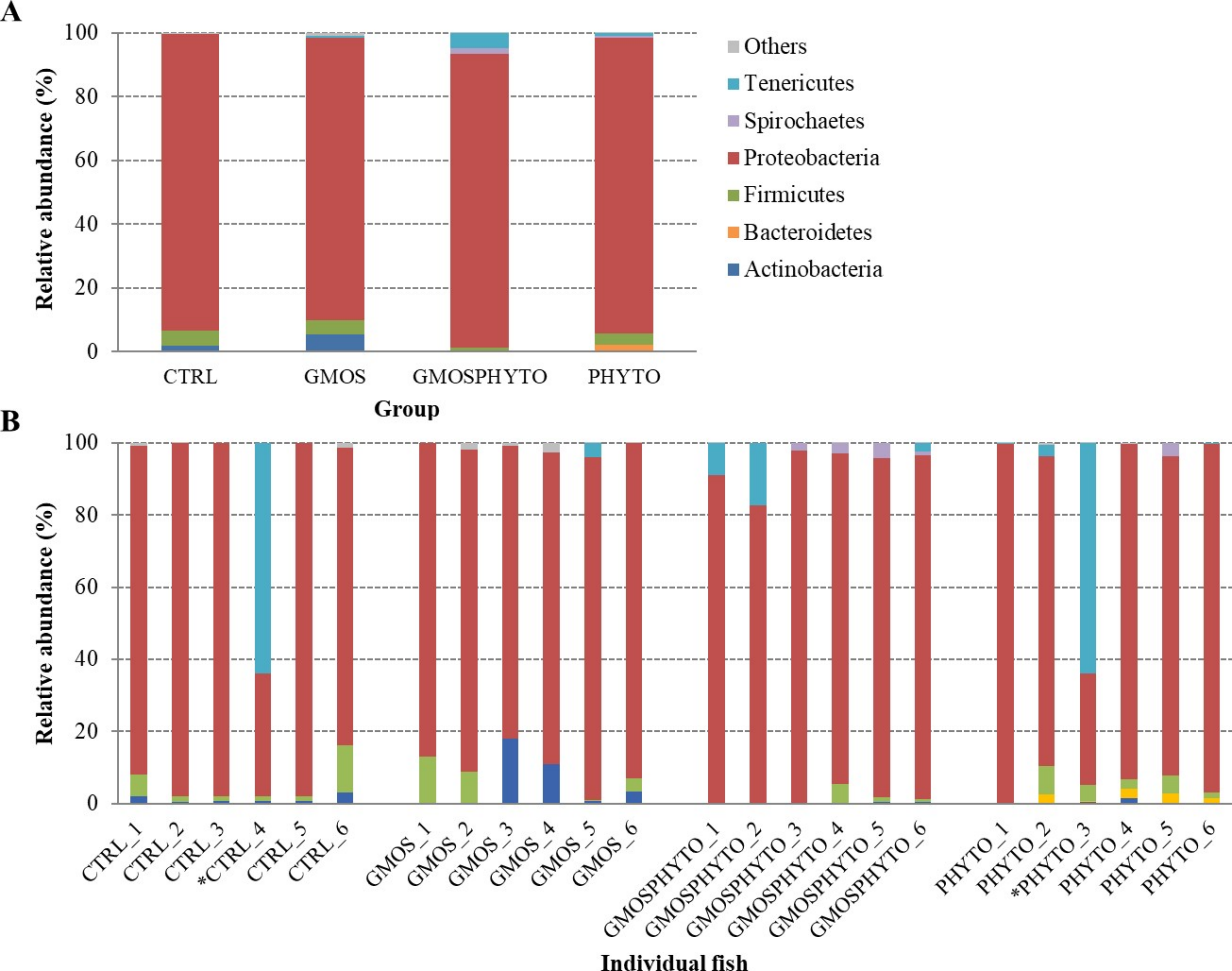
Relative abundance (%) of the most prevalent allochthonous bacterial phyla in each dietary group (A) and in individual fish (B). In the figure, all taxa with an overall abundance of ≥1% were reported. * indicates outlier samples excluded from relative abundance analysis.

**Fig 3:**
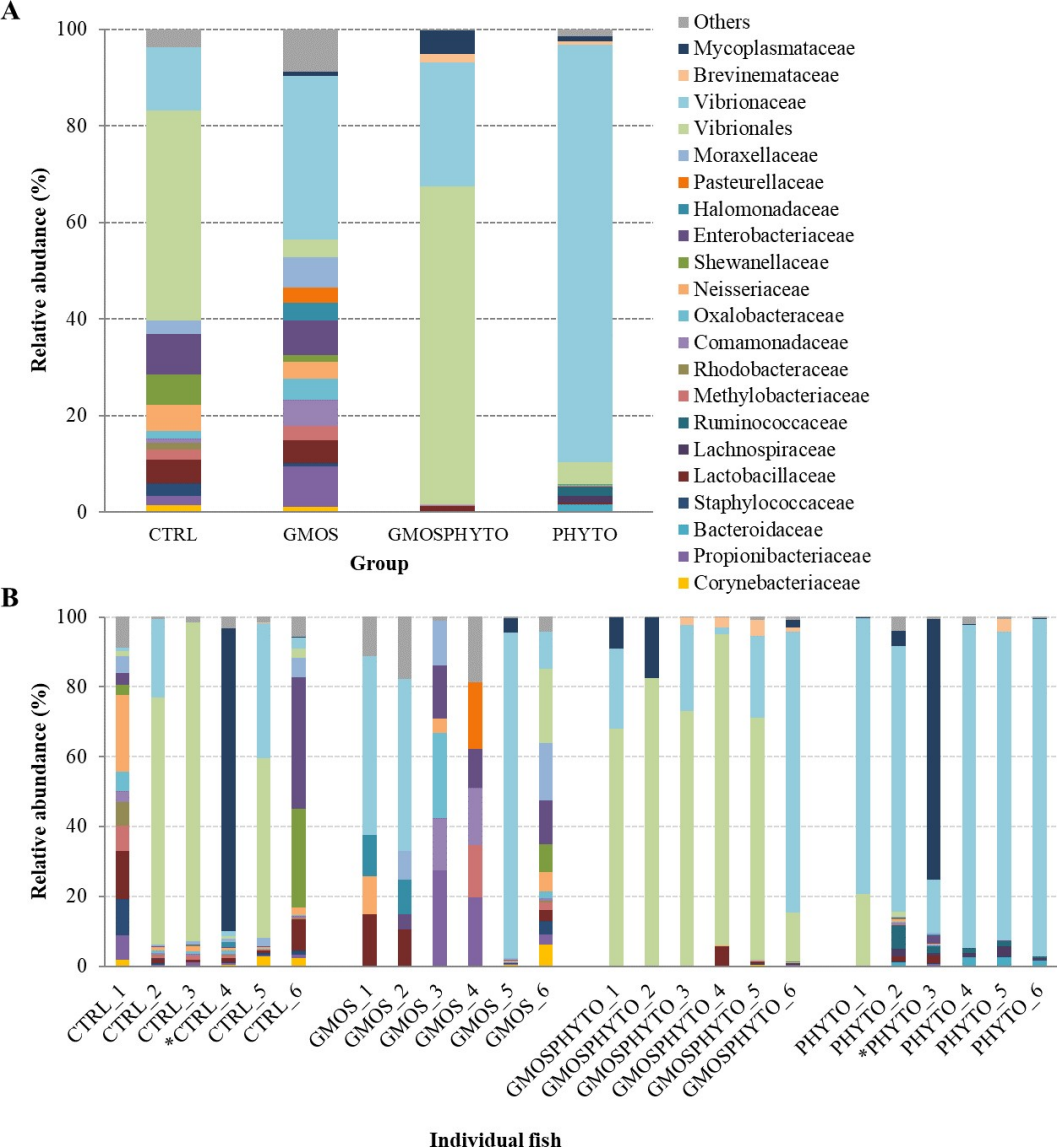
Relative abundance (%) of the most prevalent allochthonous bacterial families in each dietary group (A) and in individual fish (B). In the figure, all taxa with an overall abundance of ≥1% were reported.

The profiles of faecal microbial communities for each feeding group and individual fish are presented at phylum and family taxonomic level in Figs 2 and 3, respectively. To elaborate alpha rarefaction analysis (alpha diversity), samples were normalized at a sequencing depth of 4,500 reads. Administration of combined functional diet GMOSPHYTO, but not GMOS and PHYTO diets, significantly decreased (p<0.05) the number of observed OTUs with respect to CTRL diet. Conversely, diet type did not affect either phylogenetic diversity (Faith PD) or entropy (Shannon and Pielou’s evenness) (Table 3).

**Table 3:** Original number of reads per group-treatment assigned to OTUs, and alpha diversity metrics values (rarefied at 4500 reads) of faecal microbial community in sea bass fed CTRL, GMOS, GMOSPHYTO, and PHYTO diets.

	FEEDING GROUPS			
Item	CTRL	GMOS	GMOSPHYTO	PHYTO
Reads	48,041 ± 14,700^a^	17,556 ± 16,463^b^	47,907 ± 9,400^a^	41,004 ± 9,138^ab^
Observed OTUs	42.83 ± 9.09^a^	33.00 ± 4.73^ab^	22.00 ± 10.94^b^	36.67 ± 17.44^ab^
Shannon	2.63 ± 0.75	3.74 ± 1.29	2.31 ± 0.33	2.30 ± 0.08
Pielou’s evenness	0.49 ± 0.12	0.75 ± 0.27	0.54 ± 0.06	0.47 ± 0.10
Faith PD	4.07 ± 0.99	3.76 ± 0.99	3.07 ± 1.50	4.31 ± 1.69

Reported data are expressed as means ± SD (n = 6). The means were compared by Kruskal-Wallis test (p<0.05). Different superscript letters on the same column indicate significant differences.

**Fig 4:**
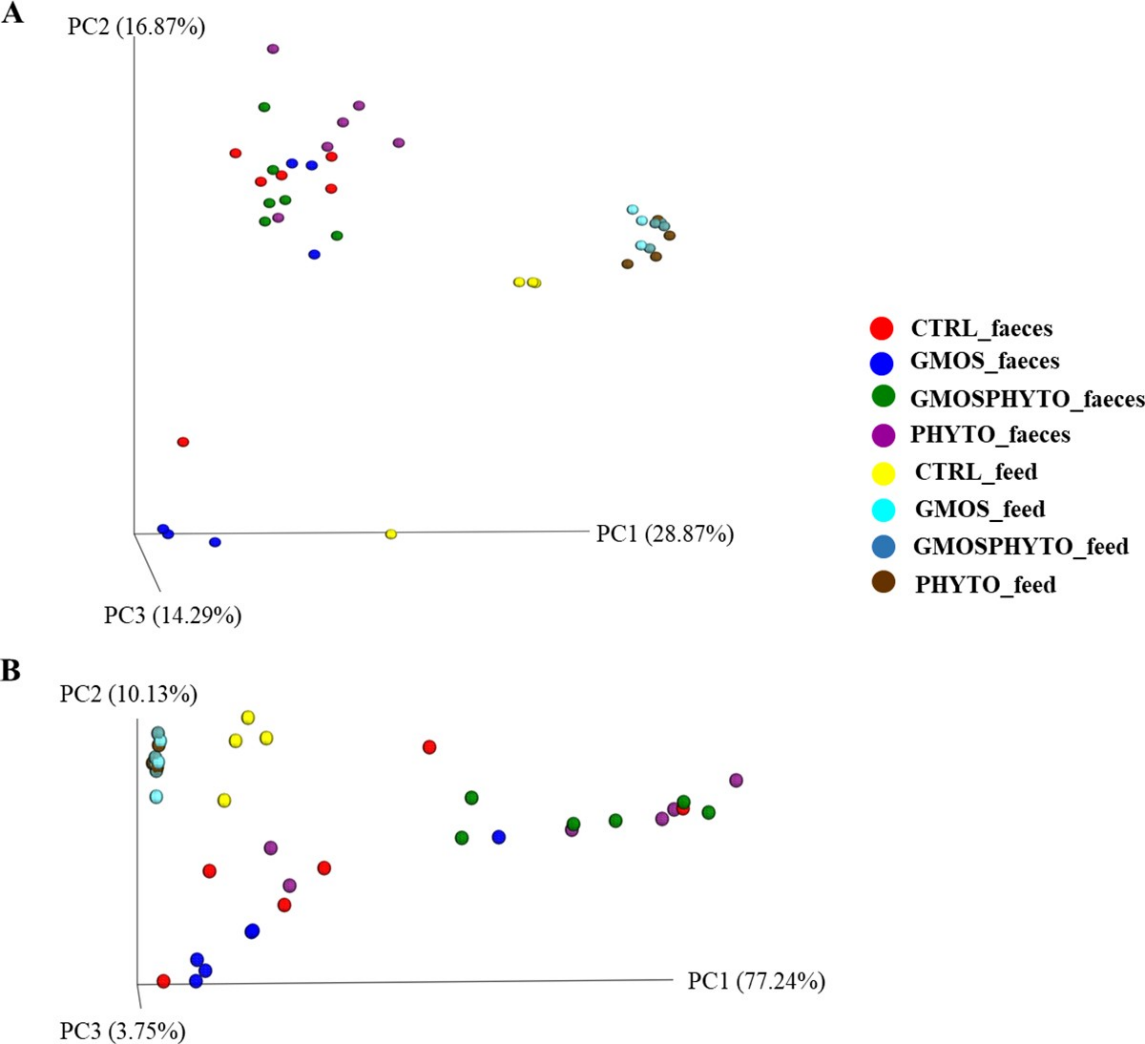
Beta diversity metrics. Principal coordinate analysis (PCoA) of Unweighted (A) and Weighted (B) Unifrac distances of gut allochthonous microbial communities associated to different diet. The figures show the 3D plot of individual fish according to their microbial profile at genus level.

Analysis of beta-diversity revealed an overall effect of diet on microbial communities both in presence/absence (unweighted UniFrac), and in relative abundance (weighted UniFrac) of OTUs (Fig 4A and 4B). However, the major effect of diet was observed in terms of relative abundance of taxa. The first principal coordinate PC1 of weighted UniFrac PCoA plot explained, indeed, 77% of the variation between individuals (Fig 4B). Interestingly, CTRL and GMOS fish groups clustered together and distinctly from PHYTO and GMOSPHYTO samples, which in turn grouped together (Fig 4B). Additionally, both in unweighted (Fig 4A) and weighted (Fig 4B) UniFrac PCoA, faecal samples appeared clearly separated from feed samples, thus indicating that observed differences between transient intestinal bacterial communities were not simply a consequence of undigested feed that might have been present in the intestinal lumen.

The permutational multivariate analysis applying Adonis and ANOSIM tests on UniFrac distance data, fully confirmed the PCoA results. Multivariate analysis on weighted data revealed significant differences between CTRL and GMOS versus PHYTO and GMOSPHYTO dietary groups (R^2^ > 0.41, R > 0.38, p<0.05). Results of pairwise comparisons on phylogenetic distances are summarized in Table 4.

**Table 4:** Results of permutational multivariate analysis of variance (Adonis) and analysis of similarity (ANOSIM) based on unweighted and weighted Unifrac distance matrices, using abundance data of faecal bacterial communities at genus level.

Adonis	Unweighted		Weighted	
	***p*-value**	**R**^**2**^	***p*-value**	**R**^**2**^
CTRL vs GMOS	0.088	0.19	0.251	0.12
CTRL vs GMOSPHYTO	**0.003**	0.22	**0.026**	0.36
CTRL vs PHYTO	**0.004**	0.30	0.131	0.18
GMOS vs GMOSPHYTO	**0.048**	0.20	**0.006**	0.68
GMOS vs PHYTO	**0.009**	0.26	**0.014**	0.41
GMOSPHYTO vs PHYTO	**0.010**	0.21	0.567	0.05
CTRL vs diet CTRL	**0.005**	0.48	0.060	0.24
GMOS vs diet GMOS	**0.030**	0.40	**0.010**	0.44
GMOSPHYTO vs diet GMOSPHYTO	**0.007**	0.48	**0.003**	0.87
PHYTO vs diet PHYTO	**0.006**	0.39	**0.009**	0.56
**ANOSIM**				
	***p*-value**	**R**	***p*-value**	**R**
CTRL vs GMOS	0.115	0.19	0.193	0.08
CTRL vs GMOSPHYTO	**0.005**	0.31	**0.028**	0.38
CTRL vs PHYTO	**0.005**	0.51	0.166	0.09
GMOS vs GMOSPHYTO	0.051	0.28	**0.007**	0.72
GMOS vs PHYTO	**0.005**	0.47	**0.018**	0.47
GMOSPHYTO vs PHYTO	**0.004**	0.39	0.260	0.03
CTRL vs diet CTRL	**0.007**	0.83	0.082	0.24
GMOS vs diet GMOS	**0.028**	0.45	**0.018**	0.53
GMOSPHYTO vs diet GMOSPHYTO	**0.002**	0.71	**0.008**	1.00
PHYTO vs diet PHYTO	**0.009**	0.50	**0.045**	0.47

Significant p-values are presented in bold.

In line with beta-diversity analysis, the major differences in terms of transient microbiota composition and taxa abundance were found between CTRL and fish feed diets containing phytogenics (i.e. PHYTO and GMOSPHYTO).

At phylum level, there were no relevant changes in transient gut microbiota profiles, which were mainly constituted by Proteobacteria, Firmicutes, and Actinobacteria phyla (Fig 2). A significant decrease (p<0.05) of bacteria belonging to the Alpha- and Betaproteobacteria classes was found in fish fed with PHYTO and GMOSPHYTO diets in comparison to control and GMOS groups that were characterized by a high number of bacteria assigned to the Rickettsiales and Neisseriales orders (Table 5). PHYTO and GMOSPHYTO dietary groups were enriched in Gammaproteobacteria essentially represented by the Vibrionales order (85–88%). Other bacterial orders belonging to Gammaproteobacteria, such as Alteromonadales, Enterobacteriales, and Pseudomonadales were practically detected only in fish fed with CTRL and GMOS diets, whereas Oceanospirillales and Pasteurellales were exclusively found in GMOS faecal samples. PHYTO were also enriched in the Clostridia class.

**Table 5:** Mean relative abundance (%) ± SD (n = 6) of the most prevalent phyla, orders, classes, families, and genera found in faecal samples of sea bass fed with four experimental diets.

TAXA	DIET											
	CTRL			GMOS			GMOSPHYTO			PHYTO		
**Phylum**												
Actinobacteria	1.84	±	1.29	5.46	±	7.41	0.13	±	0.19	0.38	±	0.66
Bacteriodetes	0.00	±	0.00	0.00	±	0.00	0.00	±	0.00	1.85	±	1.14
Firmicutes	4.82	±	4.94	4.32	±	5.46	1.27	±	2.01	3.35	±	3.08
Proteobacteria	92.84	±	6.45	88.63	±	4.90	92.12	±	5.26	92.7	±	5.63
Spirochaetes	0.03	±	0.08	0.00	±	0.00	1.71	±	1.63	0.75	±	1.67
Tenericutes	0.04	±	0.08	0.67	±	1.65	4.75	±	7.10	0.77	±	1.32
**Class** Actinobacteria	1.84	±	1.29	5.45	±	7.40	0.00	±	0.00	0.37	±	0.66
Bacteroidia	0.00	±	0.00	0.00	±	0.00	0.00	±	0.00	1.85	±	1.13
Bacilli	4.73	±	4.89	4.27	±	5.46	1.17	±	2.02	0.35	±	0.61
Clostridia	0.08	±	0.05^ab^	0.04	±	0.09^b^	0.10	±	0.15^ab^	2.97	±	2.57^a^
Alphaproteobacteria	30.71	±	32.38^ab^	35.51	±	25.31^a^	3.85	±	3.29^b^	6.45	±	12.44^b^
Betaproteobacteria	2.73	±	2.35^a^	8.40	±	10.60^a^	0.12	±	0.13^b^	0.21	±	0.46^b^
Gammaproteobacteria	59.34	±	34.08^ab^	47.70	±	31.35^b^	88.14	±	5.51^a^	86.04	±	17.08^a^
[Brevinematae]	0.00	±	0.00	0.00	±	0.00	1.70	±	1.62	0.74	±	1.67
Mollicutes	0.03	±	0.08	0.67	±	1.64	4.74	±	7.09	0.77	±	1.32
**Order**												
Actinomycetales1.84±1.29^a^5.45±7.40^ab^0.12±0.18^b^0.05±0.07^b^Bacteroidales	0.00	±	0.00	0.00	±	0.00	0.00	±	0.00	1.85	±	1.13
Bacillales	1.44	±	1.28	0.27	±	0.55	0.04	±	0.07	0.06	±	0.11
Lactobacillales	3.28	±	3.83	4.00	±	5.57	1.13	±	2.03	0.29	±	0.50
Clostridiales	0.08	±	0.05^ab^	0.04	±	0.09^b^	0.09	±	0.15^ab^	2.98	±	2.57^a^
Rhizobiales	0.86	±	0.49^a^	2.82	±	6.38^ab^	0.04	±	0.03^b^	0.06	±	0.13^b^
Rickettsiales	29.40	±	31.34^ab^	29.62	±	22.08^a^	3.80	±	3.26^b^	6.39	±	12.31^b^
Burkholderiales	0.91	±	0.63^a^	5.97	±	10.27^ab^	0.03	±	0.02^b^	0.11	±	0.23^b^
Neisseriales	1.82	±	1.78^a^	2.43	±	3.69^ab^	0.01	±	0.11^b^	0.10	±	0.23^b^
Alteromonadales	5.53	±	11.95	2.15	±	3.99	0.00	±	0.00	0.02	±	0.04
Enterobacteriales	7.36	±	15.87^a^	4.06	±	3.80^ab^	0.00	±	0.00	0.03	±	0.04^b^
Oceanospirillales	0.00	±	0.00	3.11	±	4.86	0.00	±	0.00	0.00	±	0.00
Pasteurellales	0.00	±	0.00	1.74	±	4.27	0.00	±	0.00	0.00	±	0.00
Pseudomonadales	2.07	±	2.16^a^	3.60	±	3.82^ab^	0.11	±	0.17^b^	0.09	±	0.13^b^
Vibrionales	44.36	±	39.70^bc^	30.89	±	34.12^c^	88.02	±	5.53^b^	85.90	±	17.30^a^
[Brevimatales]	0.03	±	0.07	0.00	±	0.00	1.71	±	1.62	0.75	±	1.67
Mycoplasmatales	0.03	±	0.08	0.67	±	1.64	4.74	±	7.09	0.77	±	1.32
**Family**												
*Corynebacteriaceae*	1.37	±	1.26	1.09	±	2.48	0.09	±	0.14	0.00	±	0.00
*Propionibacteriaceae*1.98±2.87^a^8.40±12.08^ab^0.04±0.05^b^0.05±0.10^b^*Bacteroidaceae*	0.00	±	0.00	0.00	±	0.00	0.00	±	0.00	1.48	±	1.09
*Bacillaceae*	0.52	±	0.49	0.00	±	0.00	0.00	±	0.00	0.02	±	0.05
*Staphylococcaceae*	2.57	±	4.40	0.68	±	1.53	0.04	±	0.07	0.06	±	0.10
*Lactobacillaceae*	5.00	±	5.96	4.78	±	6.41	1.14	±	2.22	0.33	±	0.63
*Streptococcaceae*	0.67	±	0.51^a^	0.39	±	0.89^b^	0.04	±	0.06^b^	0.05	±	0.08^b^
*Lachnospiraceae*	0.00	±	0.00	0.00	±	0.00	0.00	±	0.00	1.43	±	1.18
*Ruminococcaceae*	0.00	±	0.00	0.00	±	0.00	0.00	±	0.00	1.95	±	2.68
*Aurantimonadaceae*	0.00	±	0.00	2.28	±	5.58	0.00	±	0.00	0.00	±	0.00
*Methylobacteriaceae*	1.99	±	2.95^a^	2.91	±	6.04^ab^	0.04	±	0.04^b^	0.08	±	0.16^b^
*Rhodobacteraceae*	1.43	±	2.93	0.05	±	0.14	0.00	±	0.00	0.00	±	0.00
*Comamonadaceae*	0.95	±	1.31^a^	5.36	±	8.01^ab^	0.02	±	0.02^b^	0.07	±	0.14^b^
*Oxalobacteraceae*	1.50	±	2.31^a^	4.38	±	9.78^ab^	0.00	±	0.00	0.08	±	0.19^b^
*Neisseriaceae*	5.43	±	9.21^a^	3.49	±	4.35^ab^	0.10	±	0.11^b^	0.14	±	0.32^b^
*Shewanellaceae*	6.26	±	12.35	1.34	±	3.27	0.00	±	0.00	0.02	±	0.06
*Enterobacteriaceae*	8.29	±	16.41^a^	7.23	±	6.64^a^	0.00	±	0.00	0.04	±	0.06^b^
*Halomonadaceae*	0.00	±	0.00	3.63	±	5.66	0.00	±	0.00	0.00	±	0.00
*Pasteurellaceae*	0.00	±	0.00	3.17	±	7.77	0.00	±	0.00	0.00	±	0.00
*Moraxellaceae*	2.85	±	2.35^a^	6.28	±	7.17^ab^	0.05	±	0.07^b^	0.11	±	0.19^b^
*Vibrionaceae*	13.08	±	17.00^b^	34.00	±	37.07^b^	25.55	±	29.10^b^	86.56	±	8.71^a^
*Brevinemataceae*	0.03	±	0.08	0.00	±	0.00	1.81	±	1.75	0.75	±	1.67
*Mycoplasmataceae*	0.04	±	0.09	0.71	±	1.74	4.82	±	7.16	1.02	±	1.87
**Genus**												
*Corynebacterium*	1.37	±	1.26	1.08	±	2.48	0.09	±	0.14	0.00	±	0.00
*Propionibacterium*	1.98	±	2.87^a^	8.40	±	12.08^ab^	0.04	±	0.05^b^	0.05	±	0.10^b^
*Bacteroides*0.00±0.000.00±0.000.00±0.001.48±1.09*Staphylococcus*	2.57	±	4.40	0.68	±	1.53	0.04	±	0.07	0.06	±	0.10
*Lactobacillus*	5.00	±	5.96	4.48	±	6.41	1.14	±	2.22	0.33	±	0.62
*Streptococcus*	0.67	±	0.51^a^	0.39	±	0.89^ab^	0.04	±	0.06^b^	0.05	±	0.08^b^
*Faecalibacterium*	0.00	±	0.00	0.00	±	0.00	0.00	±	0.00	1.10	±	1.17
*Ruminococcus*	0.00	±	0.00	0.00	±	0.00	0.00	±	0.00	0.69	±	1.30
*Methylobacterium*	1.99	±	2.95^a^	2.91	±	6.04^ab^	0.04	±	0.04^b^	0.08	±	0.17^b^
*Paracoccus*	1.21	±	2.58	0.06	±	0.14	0.00	±	0.00	0.00	±	0.00
*Aquabacterium*	0.86	±	1.37	5.36	±	8.00	0.02	±	0.02	0.07	±	0.14
*Janthinobacterium*	1.37	±	2.20^a^	0.22	±	0.53^b^	0.00	±	0.00	0.08	±	0.19^b^
*Glaciecola*	0.00	±	0.00	1.88	±	4.61	0.00	±	0.00	0.00	±	0.00
*Shewanella*	6.26	±	12.35	1.34	±	3.27	0.00	±	0.00	0.02	±	0.05
*Escherichia*	8.28	±	16.39^a^	7.23	±	6.63^a^	0.00	±	0.00	0.03	±	0.06^b^
*Cobetia*	0.00	±	0.00	3.63	±	5.66	0.00	±	0.00	0.00	±	0.00
*Aggregatibacter*	0.00	±	0.00	3.17	±	7.77	0.00	±	0.00	0.00	±	0.00
*Acinetobacter*	0.95	±	1.79	0.11	±	0.28	0.00	±	0.00	0.01	±	0.02
*Enhydrobacter*	1.90	±	1.73	4.81	±	7.30	0.05	±	0.07	0.07	±	0.16
*Psychrobacter*	0.00	±	0.00	1.35	±	3.30	0.00	±	0.00	0.02	±	0.03
*Enterovibrio*	0.00	±	0.00	0.01	±	0.02	0.02	±	0.50	3.72	±	8.32
*Photobacterium*	0.65	±	0.96	17.75	±	25.22	0.22	±	0.50	18.69	±	36.78
*Vibrio*	7.82	±	10.62	0.74	±	1.81	12.92	±	11.90	30.00	±	42.93
*Mycoplasma*	0.04	±	0.08	0.71	±	1.74	4.81	±	7.16	1.02	±	1.87

Means in the same row with different letters indicate statistical significance between taxonomic groups’ abundances (p<0.05)

At family level changes in taxa abundance practically reflected what was observed at higher taxonomic levels. Interestingly, *Propionibacteriaceae*, represented by the *Propionibacterium* genus, were negatively affected by phytogenics containing diets. Similarly, the number of lactic acid bacteria assigned to the *Streptococcaceae* family was significantly reduced by all functional diets (Table 5).

When the analysis was performed at genus taxonomic level, only few genera resulted significantly influenced by diet. Among them *Streptococcus* and *Janthinobacterium* were abundant in control samples, whereas *Propionibacterium* and *Methylobacterium* in CTRL and GMOS samples. The *Bacteroides*, *Faecalibacterium* and *Ruminococcus* genera were present only in PHYTO sample. Lastly, PHYTO and GMOSPHYTO diets seemed to have a bactericidal activity against the genus *Escherichia*.

### Microbial profile and dietary modulation of autochtonous gut communities

**Fig 5:**
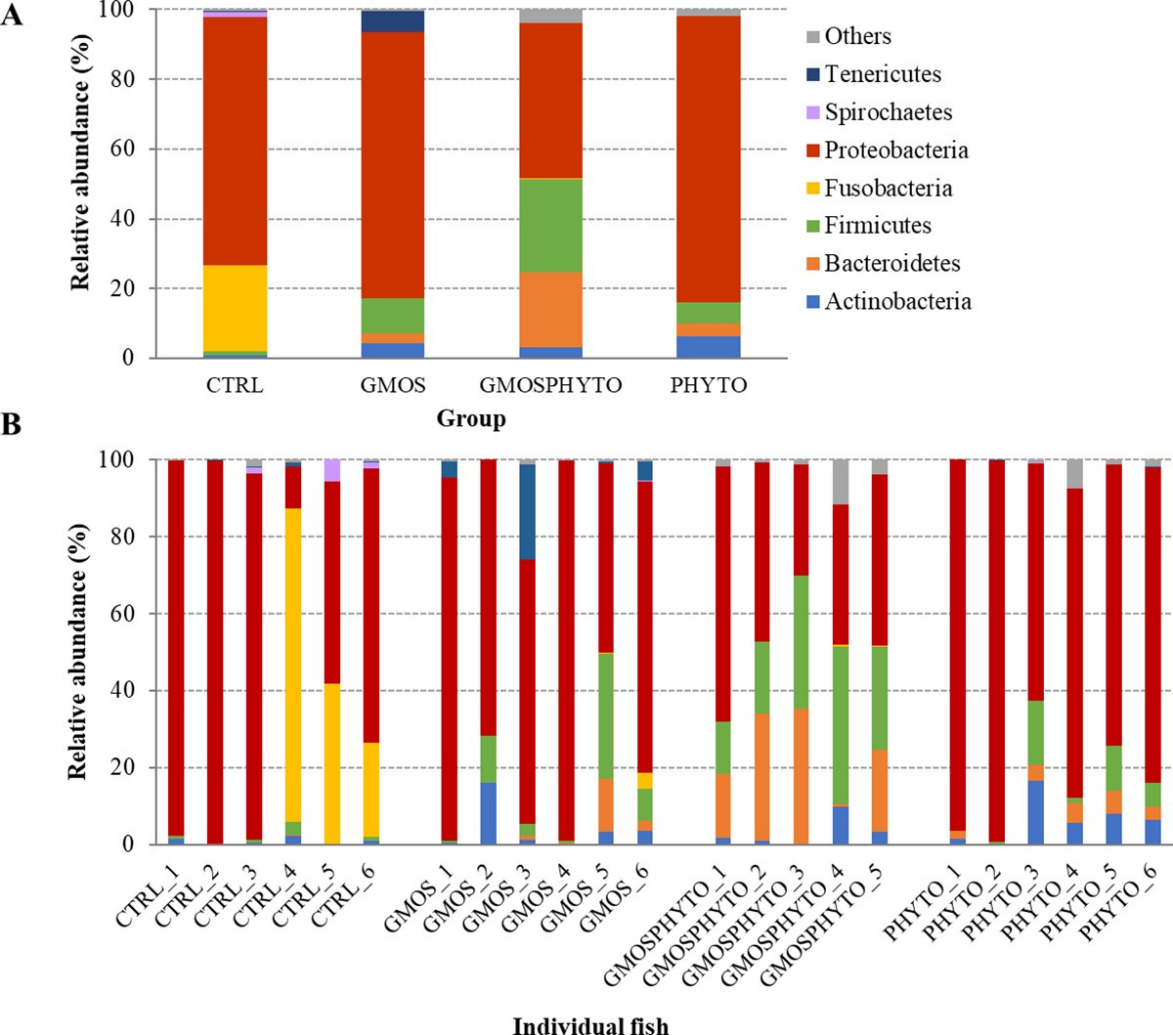
Relative abundance (%) of the most prevalent autochthonous bacterial phyla in each dietary groups (A) and in individual fish (B). In the figure, all taxa with an overall abundance of ≥1% were reported.

**Fig 6:**
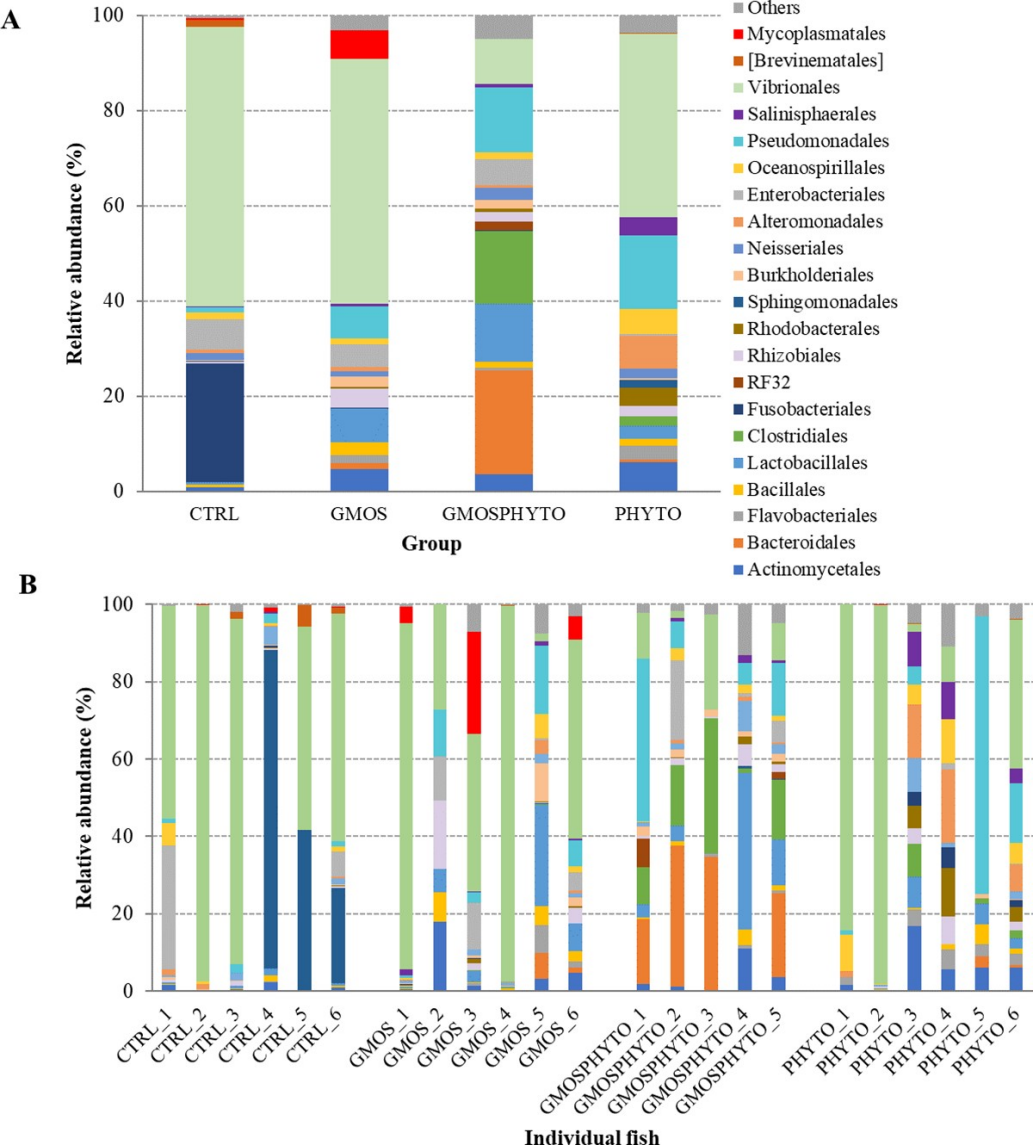
Relative abundance (%) of the most prevalent autochthonous bacterial orders in each dietary groups (A) and in individual fish (B). In the figure, all taxa with an overall abundance of ≥1% were reported.

After removing the reads corresponding to eukaryotic sequences, the entire microbial community profile of 23 intestinal mucosal samples consisted of 10 phyla, 21 classes, 40 orders, 75 families, and 112 genera. However, if we considered only the most representative taxa, the overall autochthonous microbiota was composed of 7 phyla (Actinobacteria, Bacteroidetes, Firmicutes, Fusobacteria, Proteobacteria, Spirochaetes, and Tenericutes), 11 classes (Actinobacteria, Bacteroidia, Flavobacteriia, Bacilli, Clostridia, Fusobacteriia, Alphaproteobacteria, Betaproteobacteria, Gammaproteobacteria, [Brevinematae], and Mollicutes), 21 orders (Actinomycetales, Bacteroidales, Flavobacteriales, Bacillales, Lactobacillales, Clostridiales, Fusobacteriales, RF32, Rhizobiales, Rhodobacterales, Sphingomonadales, Burkholderiales, Neisseriales, Alteromonadales, Enterobacteriales, Oceanospirillales, Pseudomonadales, Salinisphaerales, Vibrionales, [Brevinematales], and Mycoplasmatales), 42 families, and 51 genera (Tables 6 and 8). Profiles of gut mucosa communities for each feeding group and individual fish are graphically presented at phylum (Fig 5) and order (Fig 6) taxonomic level.

**Table 6:** Original number of reads per group-treatment assigned to OTUs, and alpha diversity metrics values (rarefied at 5000 reads) of mucosa microbial community in sea bass fed CTRL, GMOS, GMOSPHYTO, and PHYTO diets.

	FEEDING GROUP			
Item	CTRL	GMOS	GMOSPHYTO	PHYTO
Reads	41,853 ± 16,194^a^	41,495 ± 19,054^a^	28,386 ± 19,972^ab^	9,792 ± 8,939^b^
Observed OTUs	44 ± 20	51 ± 10	60 ± 22	41 ± 16
Shannon	2.62 ± 0.68^b^	3.35 ± 1.57^ab^	3.40 ± 1.27^ab^	4.57 ± 1.12^a^
Pielou’s evenness	0.50 ± 0.14^b^	0.55 ± 0.23^b^	0.58 ± 0.21^ab^	0.86 ± 0.16^a^
Faith PD	9.68 ± 4.46^ab^	10.34 ± 4.74^ab^	12.04 ± 4.37^a^	4.98 ±3.12^b^

Reported data are expressed as means ± SD (n = 6). The means were compared by Kruskal-Wallis test (p<0.05). Different superscript letters on the same column indicate significant differences.

To elaborate alpha rarefaction analysis (alpha diversity), samples were rarefied at 5,000 reads. Albeit, PHYTO samples had the lowest number of reads, the autochthonous gut microbial communities of fish fed with this diet were characterized by higher biodiversity than control and GMOS dietary groups, as indicated by high value of Shannon diversity index. Contrariwise, functional diets did not affected species richness (Observed OTUs) (Tables 6 and 7).

**Table 7:** Results of permutational multivariate analysis of variance (Adonis), and analysis of similarity (ANOSIM) based on unweighted and weighted UniFrac distance matrices using abundance data of intestinal mucosa-associated bacterial communities.

Adonis	Unweighted		Weighted	
	***p*-value**	**R**^**2**^	***p*-value**	**R**^**2**^
CTRL vs GMOS	0.854	0.04	0.400	0.09
CTRL vs GMOSPHYTO	0.385	0.10	0.145	0.16
CTRL vs PHYTO	**0.043**	0.19	0.314	0.10
GMOS vs GMOSPHYTO	0.374	0.10	0.506	0.07
GMOS vs PHYTO	0.136	0.13	0.263	0.11
GMOSPHYTO vs PHYTO	**0.033**	0.21	0.221	0.14
CTRL vs diet CTRL	**0.003**	0.59	**0.031**	0.38
GMOS vs diet GMOS	**0.017**	0.45	0.060	0.27
GMOSPHYTO vs diet GMOSPHYTO	**0.007**	0.67	**0.024**	0.36
PHYTO vs diet PHYTO	**0.004**	0.33	**0.018**	0.65
**ANOSIM**						
	***p*-value**	**R**	***p*-value**	**R**
CTRL vs GMOS	0.697	-0.06	0.317	0.01
CTRL vs GMOSPHYTO	0.255	0.05	0.121	0.154
CTRL vs PHYTO	**0.042**	0.28	0.289	0.03
GMOS vs GMOSPHYTO	0.488	-0.01	0.394	-0.01
GMOS vs PHYTO	0.125	0.13	0.216	0.07
GMOSPHYTO vs PHYTO	**0.042**	0.30	0.121	0.16
CTRL vs diet CTRL	**0.011**	0.79	0.081	0.34
GMOS vs diet GMOS	**0.037**	0.54	**0.215**	0.05
GMOSPHYTO vs diet GMOSPHYTO	**0.006**	1.00	**0.067**	0.33
PHYTO vs diet PHYTO	**0.033**	0.39	**0.018**	0.65

Significant p-values are in bold.

**Fig 7:**
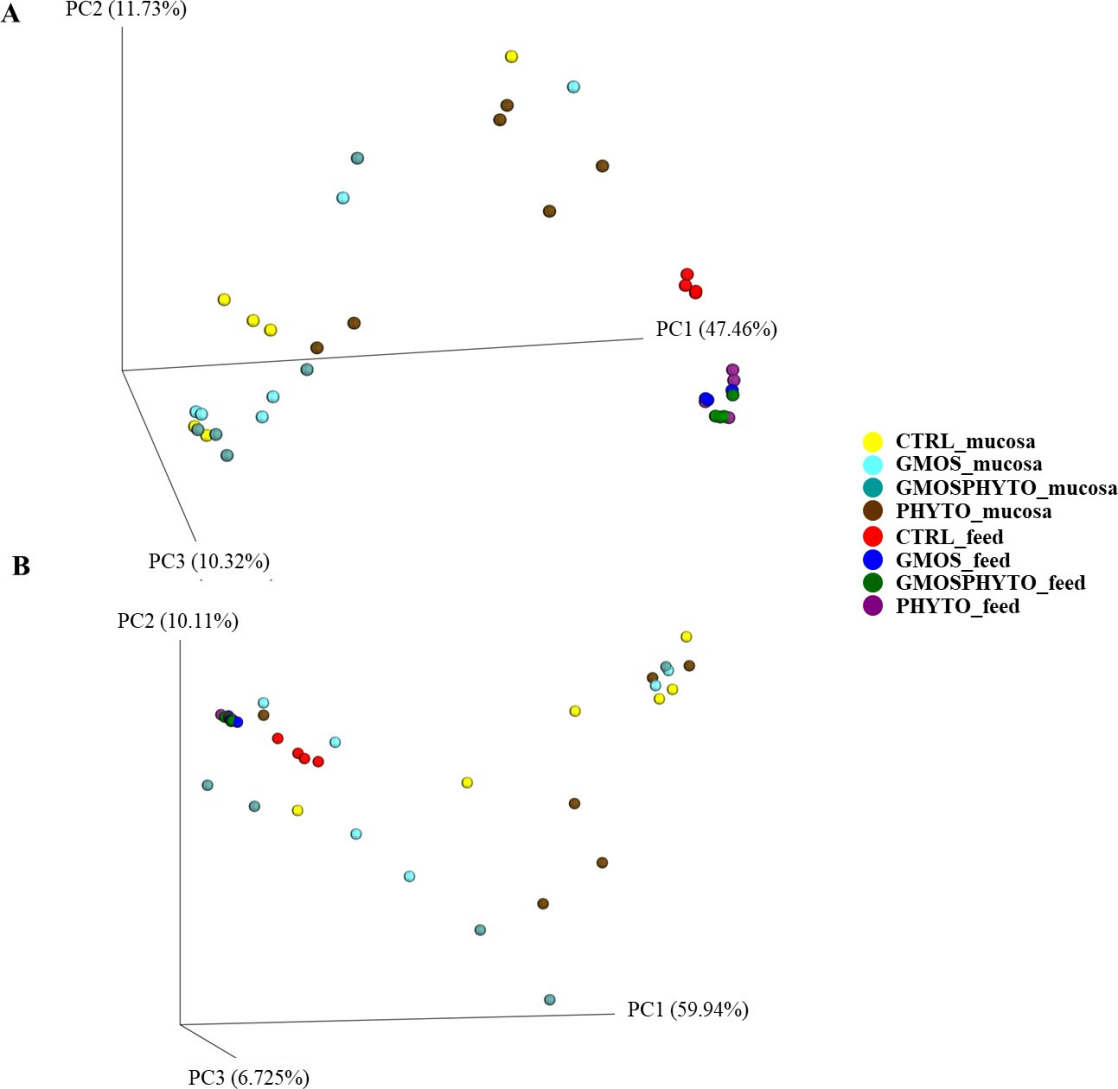
Beta diversity metrics. Principal coordinate analysis (PCoA) of Unweighted (A) and Weighted (B) Unifrac distances of gut autochthonous microbial communities associated to different diet. The figures show the 3D plot of individual fish according to their microbial profile at genus level.

Both weighted and unweighted UniFrac analyses were performed to measure microbial community diversity (beta-diversity). Data of UniFrac matrices were projected onto three-dimensional plots using principal coordinates analysis (PCoA) (Fig 7). There was a diet effect on unweighted UniFrac distances, i.e. on the presence/absence of specific taxa. Indeed, unweighted UniFrac PCoA revealed a clustering of PHYTO samples, which grouped separately from CTRL and GMOSPHYTO fish along the first principal coordinate PC1 (47% of the variation) (Fig 7A). On the contrary, in weighted PCoA, most samples were broadly indistinguishable (Fig 7B).

Likewise, for faecal bacterial communities, microbial profiles of feeds and mucosa differed to each other, both qualitatively and qualitatively. Multivariate analysis on UniFrac distance data confirmed PCoA results, indicating a significant divergence between feeding groups for only unweighted UniFrac distance matrix. Pairwise test Adonis and ANOSIM on the unweighted UniFrac data showed that fish fed diet PHYTO significantly differed (p<0.05) from CTRL (R^2^ = 0.19, R = 0.28) and GMOSPHYTO (R^2^ = 0.21, R = 0.30) (Table 6).

The variation of resident gut microbiota induced by diet was definitely lower than the variation of transient intestinal microbiota. The mucosa adhered microbial community of sea bass was mainly dominated, regardless of the diet, by three phyla: Proteobacteria, Firmicutes and Bacteroidetes (Fig 5, Table 8).

**Table 8:** Mean relative abundance (%) ± SD (n = 6) of the most prevalent phyla, orders, classes, families, and genera found in gut mucosa samples of sea bass fed with four experimental diets.

TAXA	DIET											
	CTRL			GMOS			GMOSPHYTO			PHYTO		
**Phylum**												
Actinobacteria	0.89	±	1.00	4.28	±	6.66	3.24	±	4.40	6.36	±	6.52
Bacteroidetes	0.05	±	0.10^c^	2.98	±	6.09^bc^	21.29	±	15.91^a^	3.53	±	2.40^ab^
Firmicutes	1.04	±	1.33^b^	9.85	±	13.56^ab^	26.89	±	12.88^a^	6.06	±	7.72^b^
Fusobacteria	24.58	±	36.47	0.06	±	0.11	0.15	±	0.24	0.00	±	0.00
Proteobacteria	71.12	±	38.85	76.47	±	20.28	44.54	±	16.18	82.05	±	15.78
Spirochaetes	1.48	±	2.50	0.00	±	0.01	0.00	±	0.00	0.06	±	0.13
Tenericutes	0.25	±	0.44	5.87	±	10.66	0.00	±	0.00	0.03	±	0.07
**Class**												
Actinobacteria	0.89	±	0.90	4.27	±	6.66	3.23	±	4.41	6.36	±	6.52
Bacteroidia	0.00	±	0.00	1.30	±	2.91	20.78	±	15.99	0.59	±	1.31
Flavobacteriia	0.05	±	0.08^b^	1.52	±	3.06^ab^	0.47	±	0.45^ab^	2.95	±	1.94^a^
Bacilli	1.03	±	1.19	9.40	±	12.72	12.02	±	18.53	4.10	±	4.89
Clostridia	0.01	±	0.01^b^	0.07	±	0.07^b^	14.76	±	14.29^a^	1.96	±	3.62^b^
Fusobacteriia	24.58	±	32.62	0.06	±	0.11	0.15	±	0.24	0.00	±	0.00
Alphaproteobacteria	1.54	±	1.72	8.79	±	11.30	10.31	±	6.89	8.38	±	11.74
Betaproteobacteria	1.68	±	1.81	3.22	±	5.00	4.11	±	2.71	2.32	±	3.52
Gammaproteobacteria	67.90	±	36.11	64.35	±	28.67	30.12	±	17.71	71.35	±	26.67
[Brevinematae]	1.48	±	2.23	0.00	±	0.01	0.00	±	0.00	0.06	±	0.13
Mollicutes	0.25	±	0.39	5.87	±	10.66	0.00	±	0.00	0.03	±	0.07
**Order**												
Actinomycetales	0.90	±	1.01	4.68	±	7.50	3.56	±	4.99	6.07	±	6.58
Bacteroidales	0.00	±	0.00	1.33	±	2.97	21.87	±	17.06	0.59	±	1.31
Flavobacteriales	0.05	±	0.09^b^	1.56	±	3.12^ab^	0.50	±	0.48^ab^	2.96	±	1.95^a^
Bacillales	0.45	±	0.61	2.70	±	3.48	1.35	±	1.76	1.40	±	2.04
Lactobacillales	0.59	±	0.75	7.16	±	10.83	12.04	±	19.15	2.73	±	3.76
Clostridiales	0.01	±	0.01^b^	0.07	±	0.08^b^	15.31	±	14.36^a^	1.98	±	3.68^b^
Fusobacteriales	24.79	±	36.87	0.06	±	0.11	0.17	±	0.27	0.00	±	0.00
RF32	0.00	±	0.00	0.00	±	0.00	1.84	±	3.68	0.00	±	0.00
Rhizobiales	0.44	±	0.42	4.02	±	7.65	2.10	±	2.37	2.26	±	3.19
Rhodobacterales	0.05	±	0.10	0.30	±	0.47	0.63	±	1.05	3.70	±	5.60
Sphingomonadales	0.07	±	0.13	0.09	±	0.08	0.00	±	0.00	1.78	±	2.52
Burkholderiales	0.26	±	0.26	2.16	±	4.27	1.92	±	0.54	0.28	±	0.55
Neisseriales	1.45	±	2.04	1.12	±	0.94	2.55	±	3.54	2.07	±	3.74
Alteromonadales	0.65	±	0.79	0.85	±	1.65	0.55	±	0.59	6.89	±	8.93
Enterobacteriales	6.42	±	14.28	4.72	±	6.32	5.40	±	10.13	0.30	±	0.66
Oceanospirillales	1.40	±	2.52	1.37	±	2.71	1.41	±	1.49	5.21	±	5.17
Pseudomonadales	1.21	±	1.18	6.72	±	7.84	13.57	±	19.14	15.51	±	31.50
Salinisphaerales	0.06	±	0.12	0.58	±	0.70	0.76	±	0.97	3.75	±	5.15
Vibrionales	58.82	±	38.47	51.33	±	40.91	9.57	±	11.37	38.67	±	48.24
[Brevinematales]	1.49	±	2.50	0.00	±	0.01	0.00	±	0.00	0.06	±	0.13
Mycoplasmatales	0.25	±	0.44	6.11	±	11.47	0.00	±	0.00	0.03	±	0.07
**Family**												
*Bogoriellaceae*	0.00	±	0.00	0.00	±	0.00	0.23	±	0.47	0.65	±	1.45
*Corynebacteriaceae*	0.04	±	0.07	0.23	±	0.38	0.17	±	0.27	1.77	±	1.83
*Micrococcaceae*	0.08	±	0.17	0.01	±	0.03	0.07	±	0.10	2.32	±	4.26
*Propionibacteriaceae*	0.70	±	0.72	4.32	±	7.65	3.09	±	4.90	1.33	±	1.25
*Bacteroidaceae*	0.00	±	0.00	0.00	±	0.00	12.07	±	13.93	0.31	±	0.70
*Porphyromonadaceae*	0.00	±	0.00	1.33	±	2.97	0.92	±	1.09	0.00	±	0.00
*Prevotellaceae*	0.00	±	0.00	0.00	±	0.00	7.50	±	14.99	0.27	±	0.61
*[Paraprevotellaceae]*	0.00	±	0.00	0.00	±	0.00	0.79	±	1.59	0.00	±	0.00
*Flavobacteriaceae*	0.00	±	0.01	0.47	±	0.86	0.47	±	0.52	2.30	±	2.38
*[Weeksellaceae]*	0.04	±	0.09	1.08	±	2.27	0.03	±	0.06	0.66	±	1.39
*Planococcaceae*	0.01	±	0.03	0.81	±	1.82	0.00	±	0.00	0.00	±	0.00
*Staphylococcaceae*	0.33	±	0.37	1.72	±	3.36	1.22	±	1.80	1.39	±	2.05
*Enterococcaceae*	0.00	±	0.01	3.68	±	8.22	0.02	±	0.04	0.39	±	0.88
*Lactobacillaceae*	0.50	±	0.65	2.74	±	2.56	11.48	±	18.53	1.21	±	1.61
*Streptococcaceae*	0.08	±	0.09	0.54	±	0.96	0.54	±	0.64	1.12	±	2.49
*Lachnospiraceae*	0.00	±	0.00	0.00	±	0.00	9.13	±	11.16	0.28	±	0.63
*Ruminococcaceae*	0.00	±	0.01	0.00	±	0.00	3.46	±	2.82	0.00	±	0.00
*Veillonellaceae*	0.00	±	0.00	0.00	±	0.00	1.97	±	2.79	0.00	±	0.00
*[Tissierellaceae]*	0.01	±	0.01	0.06	±	0.05	0.21	±	0.25	1.70	±	3.79
*Fusobacteriaceae*	24.79	±	36.87	0.06	±	0.11	0.17	±	0.27	0.00	±	0.00
*Methylobacteriaceae*	0.37	±	0.38	3.93	±	7.69	1.59	±	1.64	1.80	±	2.39
*Rhodobacteraceae*	0.05	±	0.10	0.30	±	0.47	0.63	±	1.05	3.70	±	5.60
*Acetobacteraceae*	0.00	±	0.00	0.93	±	2.08	0.00	±	0.00	0.34	±	0.76
*Erythrobacteraceae*	0.07	±	0.13	0.09	±	0.08	0.00	±	0.00	1.08	±	1.57
*Sphingomonadaceae*	0.00	±	0.00	0.00	±	0.00	0.00	±	0.00	0.71	±	0.97
*Alcaligenaceae*	0.06	±	0.05	0.18	±	0.35	1.52	±	0.66	0.00	±	0.00
*Comamonadaceae*	0.07	±	0.10	1.85	±	3.98	0.17	±	0.18	0.26	±	0.56
*Neisseriaceae*	1.45	±	2.04	1.12	±	0.94	2.55	±	3.54	2.07	±	3.74
*Alteromonadaceae*	0.47	±	0.64	0.56	±	1.12	0.37	±	0.47	4.47	±	5.52
*Idiomarinaceae*	0.01	±	0.02	0.28	±	0.53	0.03	±	0.04	2.43	±	3.43
*Enterobacteriaceae*	6.42	±	14.28	4.72	±	6.32	5.40	±	10.13	0.30	±	0.66
*Alcanivoracaceae*	0.00	±	0.00	0.02	±	0.02	0.00	±	0.00	1.33	±	1.85
*Halomonadaceae*	1.40	±	2.52	1.36	±	2.71	1.41	±	1.49	3.88	±	3.89
*Moraxellaceae*	1.15	±	1.09	6.69	±	7.88	3.06	±	3.12	1.20	±	1.20
*Pseudomonadaceae*	0.07	±	0.13	0.04	±	0.05	10.51	±	20.20	14.31	±	30.93
*Salinisphaeraceae*	0.06	±	0.12	0.58	±	0.70	0.76	±	0.97	3.75	±	5.15
*Piscirickettsiaceae*	0.00	±	0.00	0.00	±	0.00	0.00	±	0.00	0.68	±	1.52
*Pseudoalteromonadaceae*	4.56	±	9.60	0.06	±	0.14	0.05	±	0.06	1.46	±	2.03
*Vibrionaceae*	30.14	±	39.52	49.76	±	42.21	8.06	±	11.39	33.82	±	46.31
*Xanthomonadaceae*	0.02	±	0.04	0.64	±	1.40	0.00	±	0.00	0.00	±	0.00
*Brevinemataceae*	1.49	±	2.50	0.00	±	0.01	0.00	±	0.00	0.06	±	0.13
*Mycoplasmataceae*	0.25	±	0.44	6.11	±	11.47	0.00	±	0.00	0.03	±	0.07
**Genus**												
*Georgenia*	0.00	±	0.00	0.00	±	0.00	0.23	±	0.47	0.65	±	1.45
*Corynebacterium*	0.04	±	0.07	0.23	±	0.38	0.17	±	0.27	1.77	±	1.83
*Kocuria*	0.05	±	0.11	0.01	±	0.03	0.02	±	0.04	0.80	±	1.79
*Rothia*	0.00	±	0.00	0.00	±	0.00	0.00	±	0.00	0.73	±	1.63
*Propionibacterium*	0.70	±	0.72	4.32	±	7.65	3.09	±	4.90	1.33	±	1.25
*Bacteroides*	0.00	±	0.00	0.00	±	0.00	12.07	±	13.93	0.31	±	0.70
*Dysgonomonas*	0.00	±	0.00	1.33	±	2.97	0.00	±	0.00	0.00	±	0.00
*Parabacteroides*	0.00	±	0.00	0.00	±	0.00	0.92	±	1.09	0.00	±	0.00
*Prevotella*	0.00	±	0.00	0.00	±	0.00	7.50	±	14.99	0.27	±	0.61
*[Prevotella]*	0.00	±	0.00	0.00	±	0.00	0.79	±	1.59	0.00	±	0.00
*Salegentibacter*	0.00	±	0.00	0.04	±	0.09	0.00	±	0.00	0.78	±	1.08
*Chryseobacterium*	0.00	±	0.00	0.00	±	0.00	0.00	±	0.00	0.63	±	1.40
*Wautersiella*	0.00	±	0.00	1.01	±	2.27	0.00	±	0.00	0.00	±	0.00
*Staphylococcus*	0.33	±	0.37	1.72	±	3.36	1.22	±	1.80	1.39	±	2.05
*Enterococcus*	0.00	±	0.01	3.38	±	7.56	0.02	±	0.04	0.00	±	0.00
*Lactobacillus*	0.50	±	0.65	2.74	±	2.56	11.44	±	18.56	0.79	±	1.10
*Streptococcus*	0.08	±	0.09	0.22	±	0.25	0.47	±	0.67	1.12	±	2.49
*Blautia*	0.00	±	0.00	0.00	±	0.00	0.79	±	0.78	0.00	±	0.00
*Lachnospira*	0.00	±	0.00	0.00	±	0.00	4.89	±	8.02	0.00	±	0.00
*Roseburia*	0.00	±	0.00	0.00	±	0.00	1.61	±	1.34	0.00	±	0.00
*Faecalibacterium*	0.00	±	0.01	0.00	±	0.00	3.25	±	2.50	0.00	±	0.00
*Dialister*	0.00	±	0.00	0.00	±	0.00	0.91	±	1.82	0.00	±	0.00
*Phascolarctobacterium*	0.00	±	0.00	0.00	±	0.00	1.06	±	1.05	0.00	±	0.00
*Anaerococcus*	0.01	±	0.01	0.05	±	0.06	0.00	±	0.01	0.82	±	1.84
*Cetobacterium*	24.79	±	36.87	0.02	±	0.05	0.02	±	0.03	0.00	±	0.00
*Methylobacterium*	0.37	±	0.38	3.93	±	7.69	1.59	±	1.64	1.80	±	2.39
*Loktanella*	0.01	±	0.03	0.02	±	0.03	0.00	±	0.00	1.05	±	2.36
*Nautella*	0.00	±	0.00	0.00	±	0.00	0.00	±	0.00	0.69	±	1.54
*Paracoccus*	0.03	±	0.07	0.22	±	0.50	0.25	±	0.43	1.29	±	1.89
*Erythrobacter*	0.07	±	0.13	0.09	±	0.08	0.00	±	0.00	1.08	±	1.57
*Sphingomonas*	0.00	±	0.00	0.00	±	0.00	0.00	±	0.00	0.71	±	0.97
*Sutterella*	0.00	±	0.00	0.00	±	0.00	1.21	±	1.00	0.00	±	0.00
*Comamonas*	0.00	±	0.00	1.80	±	4.01	0.00	±	0.00	0.00	±	0.00
*Alteromonas*	0.15	±	0.26	0.01	±	0.02	0.00	±	0.00	0.90	±	1.32
*Glaciecola*	0.31	±	0.58	0.02	±	0.03	0.00	±	0.00	0.76	±	1.04
*Marinobacter*	0.01	±	0.02	0.49	±	0.99	0.37	±	0.47	2.81	±	3.46
*Idiomarina*	0.01	±	0.02	0.28	±	0.53	0.03	±	0.04	2.43	±	3.43
*Escherichia*	6.42	±	14.28	4.67	±	6.36	5.40	±	10.13	0.30	±	0.66
*Alcanivorax*	0.00	±	0.00	0.02	±	0.02	0.00	±	0.00	1.33	±	1.85
*Chromohalobacter*	0.02	±	0.05	0.02	±	0.05	0.22	±	0.31	0.82	±	1.12
*Cobetia*	1.28	±	2.58	0.01	±	0.01	0.00	±	0.00	0.00	±	0.00
*Halomonas*	0.07	±	0.15	1.32	±	2.65	1.19	±	1.37	2.30	±	2.51
*Acinetobacter*	0.17	±	0.39	3.56	±	7.80	0.18	±	0.32	0.68	±	0.98
*Enhydrobacter*	0.50	±	0.95	2.97	±	5.23	2.32	±	3.07	0.52	±	0.70
*Pseudomonas*	0.07	±	0.13	0.04	±	0.05	10.51	±	20.20	10.62	±	22.68
*Salinisphaera*	0.06	±	0.12	0.51	±	0.62	0.49	±	0.69	3.75	±	5.15
*Pseudoalteromonas*	4.56	±	9.60	0.06	±	0.14	0.05	±	0.06	1.46	±	2.03
*Photobacterium*	6.09	±	13.39	0.50	±	0.72	0.09	±	0.18	14.94	±	33.41
*Vibrio*	1.74	±	3.89	0.04	±	0.05	0.30	±	0.61	16.80	±	37.56
*Stenotrophomonas*	0.00	±	0.00	0.63	±	1.41	0.00	±	0.00	0.00	±	0.00
*Mycoplasma*	0.25	±	0.44	6.11	±	11.47	0.00	±	0.00	0.03	±	0.07

Means in the same row with different letters indicate statistical significance between taxonomic groups’ abundances (p<0.05).

Among them, the amount of Firmicutes was positively influenced (p<0.05) by administration of GMOSPHYTO diet (Table 8). This was essentially due to the enrichment in bacteria belonging to the Clostridiales order of the Clostridia class. No differences in relative abundance were found at lower taxonomic level, however several families and genera seemed to have a diet-specific association. The *Prevotellaceae*, *Ruminococcaceae*, *Bacteroidaceae*, and *Veillonellaceae* families were identified only in fish fed diet GMOSPHYTO, whereas *Fusobacteriaceae* were found solely in intestine of control feeding group (Table 8).

Accordingly, the orders Clostridiales, Lactobacillales and Pseudomonadales, as well as the *Bacteroides* and *Prevotella* genera were particularly abundant in resident gut microbiota of fish fed with GMOSPHYTO diet. Similarly, in the same fish group, several genera of Clostridiales, such as *Blautia*, *Lachnospira*, *Roseburia*, *Faecalibacterium*, *Dialister*, and *Phascolarctobacterium*, were identified. On the contrary, *Cetobacterium*, belonging to the *Fusobacteriaceae* family, was found only in the autochthonous gut microbiota of control fish (Table 8).

## Discussion

Improving fish health is a major concern for fish farmers. Since 2006 when a European Union-wide ban on the use of antibiotics as growth promoters in animal feed entered into effect (EU Regulation No. 1831/2003), functional feeds with selected additives such as probiotics, prebiotics and phytogenics started to be used in aquaculture and other animal production industries to promote fish health [55].

In our study, the choice to use functional feed additives, such as galactomannan oligosaccharides (GMOS) from mucilage and phytogenics in low FM/FO diets, meets the consumer’s demand of eco-friendly production practices. European sea bass (*Dicentrarchus labrax*) promptly accepted all the experimental diets for all the duration of the feeding trial, but growth performance, feed intake and feed efficiency were unaffected by prebiotic and/or phytogenic incorporation [20].

Marker gene analysis of 16S rRNA was successfully applied to the study of both gut mucosa-associated (autochthonous) and lumen content (allochthonous) microbial communities. The analysis revealed that autochthonous microbiota was characterized by higher species richness than allochthonous microbiota. Number of bacterial genera observed in mucosa samples was indeed, higher than in faecal samples, however autochthonous microbiota was rarefied to a higher read count. Consistent with our results, similar differences in bacterial abundance between transient and mucosa-associated gut microbiota have been previously described in sea bass using 16S rRNA gene sequencing [56], as well as in other species [57–59, 48].

As reported in literature, Proteobacteria resulted the most common bacteria phylum in both transient and resident microbial communities of sea bass intestine irrespective to the diet [56, 60]. No significant differences on allochthonous bacterial alpha-diversity and species richness were observed in response to diets containing functional ingredients. In contrast, the mucosa-associated microbiota of fish fed with PHYTO diet, supplemented with a mixture of garlic and labiate-plants oils, showed an increase of Shannon diversity index in comparison to the control feeding group. Carda-Diéguez and colleagues [56] did not find any differences in terms of autochthonous microbiota biodiversity in sea bass fed a functional diet containing β-glucans and essential oils, whereas in rainbow trout (*Oncorhynchus mykiss*), intestinal resident bacterial diversity and richness were negatively affected by garlic extract in the diet [61]. Nevertheless, in general, the lack of a negative effect on microbial diversity and species richness, like in our study, are considered desired features. High intestinal microbial biodiversity usually reflects a healthy status of the host, because a reduction in commensal bacterial diversity and/or species richness may result in diminished colonization resistance against incoming opportunistic pathogens, which have the potential to induce infection of the intestinal tract [62–63].

Although transient and resident gut microbiota of sea bass was dominated by the same phyla (Proteobacteria, Firmicutes, Actinobacteria) irrespective to the diet, the present study clearly showed that gut microbiota can be modulated by dietary supplementation with functional ingredients.

Prebiotics, such as mannan oligosaccharides (MOS) and phytogenics have proved to be effective at modulating fish microbiota in several studies [64, 18, 46, 56, 65, 12, 61]. In the present study, allochthonous intestinal microbiota of sea bass fed GMOS diet, but not GMOSPHYTO, showed reduced number of Gammaproteobacteria, mainly represented by the Vibrionales order. This could be considered a positive effect of dietary GMOS administration, since the Vibrionales order includes several potentially pathogenic species for fish, such as *Vibrio anguillarum*, and *Photobacterium damselae*. Similarly, Guardiola et al. [66] showed in sea bream that dietary administration of fenugreek (*Trigonella feonum-graecum* L.) seeds, combined with probiotics strains, for 3 weeks, increased skin mucus bactericidal activity against the pathogenic bacterium *P*. *damselae* in comparison to fish of the control and other experimental groups. Seeds of fenugreek are a rich source of a wide variety of nutritive and bioactive compounds, including galactomannan, having several potent activities including antimicrobial and anti-inflammatory effects.

Accordingly, our tested diets with low levels of ingredients of marine origin, and supplemented with GMOS and PHYTO improved significantly European sea bass resistance to *V*. *anguillarum* after intestinal infection and stress challenge [20]. However, although the three functional diets reduced gut pathogen translocation rate, there was an evident greater protective effect when GMOS or PHYTO were supplemented to the diet individually. Indeed, the relative percentage of survival was 33%, 47%, 20%, and 40% for fish fed GMOS, PHYTO, GMOSPHYTO, and CTRL diets, respectively [20]. Furthermore, at morphology level, the combination of two additives, GMOS and PHYTO, invalidated their site-specific anti-inflammatory properties on fish intestine [20]. This observed effectiveness reduction could indicate a possible antagonistic effect between products, effect that appeared to be confirmed by our 16S analysis of gut microbial communities, which showed an increased amount of Vibrionales when GMOS and PHYTO were added together to the feed, but not when only GMOS was supplemented to the diet.

Similarly, previous evidences in European sea bass revealed a positive effect of prebiotic mannan-oligosaccharides (MOS) in terms of fish survival to *V*. *anguillarum* and *V*. *alginolyticus* infection [22, 67]. Also, juvenile trout fed with a diet supplemented with 0.2% MOS showed a significantly decrease of *Aeromonas*/*Vibrio* spp. in comparison to the control group [64]. MOS is able to bind the threadlike fimbriae of gram-negative pathogenic bacteria (i.e. *Aeromonas*, *Vibrio*, and *Pseudomonas*) preventing them from attaching to the gut wall thus avoiding their intestinal colonization [68]. Dietary inclusion of MOS caused also an enhancement in the number of cells secreting acid mucins in posterior gut of fish [25, 26]. Mucins are the major antiadhesive components of mucus and the improvement in mucus secretion represents, therefore, the primary defence mechanism against colonizing organisms of gut epithelial mucosal surface.

Bacteria assigned to the Clostridiales order were more abundant in intestinal content of fish fed PHYTO diet, as well as, in mucosa-associated microbiota of fish receiving GMOSPHYTO diet. In line with our results, a significant and positive correlation was found in resident intestinal microbiota of rainbow trout between the abundance of the genus *Clostridium* and the levels of garlic oil in the diet [61]. Previous studies have highlighted the importance of some members of the Clostridiales order, which includes several butyrate producers. It is known, indeed, that members of the genus *Clostridium*, besides being butyrogenic, can improve fish nutrition by providing essential fatty acids and vitamins [69, 70, 71]. Accordingly, among Clostridiales, the *Faecalibacterium* and *Ruminococcus* genera were found only in the intestinal lumen of fish fed PHYTO diet. In particular, the *Ruminococcus* genus plays an important role in the degradation of indigestible carbohydrate, such as resistant starch and dietary fibers, thus contributing to the more efficient energy utilization of feed and to intestinal health of host by producing butyrate as end product of dietary fiber fermentation [72, 73, 74]. Similarly, *Faecalibacterium* is the most important commensal butyrate-producing bacterium in the human colon and it is considered as a bio-indicator of human health [75].

Interestingly, dietary supplementation with phytogenics inhibited coliform bacteria; specifically *Escherichia* genus was practically undetectable in allochthonous microbiota of fish fed PHYTO and GMOSPHYTO diets. Accordingly, a reduction in Coliforms and in *Escherichia coli* counts were reported in the cecum, small and large intestine of broilers fed with diets containing phytogenic feed additives [76–78].

In the last two decades EOs have been extensively investigated for their use as feed additives for terrestrial animals [79, 34]; only limited information is instead available about their potential benefits on fish health [41]. Studies reporting the effect of dietary EOs on gut bacterial composition of fish are indeed, still scarce, therefore our study represents a contribution. Consistent with our results, Ran et al. [65] tested the effects of a commercial blend of thymol and carvacrol essential oils in hybrid tilapia finding a reduction of *E*. *coli*/Coliform in the gut microbiota. Similarly, in another study in tilapia (*Oreochromis niloticus*), supplementation of diet with EOs from lemongrass, and geranium significantly decreased intestinal total bacteria, Coliforms, *E*. *coli*, and *Aeromonas* counts [12].

Numerous studies have demonstrated the antimicrobial effects of EOs *in vitro*, too. Among these, Helander et al. [80] demonstrated bactericidal properties of EOs from oregano, carvacrol, and thymol versus *E*. *coli* and *Salmonella*, whereas Ankri and Mirelman [81] described the wide spectrum antibacterial activity of garlic against Gram-negative and Gram-positive bacteria, including *E*. *coli*. The antimicrobial mechanism of essential oils is related to their lipophilic properties and chemical composition. EOs can damage bacteria cells affecting both, the membrane and cytoplasm. Indeed, the lipophilic nature of EO compounds allows them to penetrate cell membrane and remain between the phospholipid bilayer thus changing bacterial membrane structure and, consequently, its permeability. EOs can also affect quorum sensing systems [82, 43]. However, although, the antimicrobial properties of EOs have been clearly evidenced either *in vitro* or *in vivo*, it is also true that the commonly used microbiome profiling methods focus on the relative abundance or proportions of OTUs, meaning that that they cannot discriminate whether an enrichment in certain species is due to an increase in absolute abundance or a decrease in the abundances of other dominant taxa.

In the present study, changes in gut mucosa-adhered (resident) microbial communities were definitely less pronounced in comparison to dietary modulation of transient gut microbiota. This is a direct consequence of the fact that feedstuffs are a major source of allochthonous bacteria, which can temporarily integrate into the gut transient microbiome. However, it does not mean that the composition of transient bacterial communities is simply a mirror of feed-borne bacteria. Indeed, microbial profile of feeds resulted different from faecal microbial profiles. Otherwise, the data shows that even though there was a very large relative abundance of LAB in the diets, they failed to colonize/establish in the gut. They were not observed in the allochthonous bacteria, whereas in the autochthonous, they only represented an important order in the GMOSPTHYTO group and to a lesser extent in the GMOS group.

At resident microbiota level, only the combination of both functional ingredients (GMOSPHYTO) caused a significant increase of Firmicutes, essentially due to the enrichment in bacteria belonging to the Clostridiales order. In addition to *Clostridium*, Firmicutes phylum includes different genera of lactic acid bacteria (LAB) such as *Streptococcus*, *Lactobacillus*, and *Leuconostoc*. LAB are generally considered beneficial microorganisms and used as probiotics for fish and for other vertebrates [83, 84]. Therefore, an increase in their number is usually considered desirable, because they are associated with a healthy intestine. However, in sea bass, the number of LAB did not increase in response to dietary administration of prebiotic GMOS. This was an unexpected result since galactomannan-oligosaccharides are complex and indigestible carbohydrates (dietary fiber) that can be used as a substrate by LAB. Previous studies using similar feed additives reported an increase in LAB number in sea bream (*Sparus aurata*), Nile tilapia (*O*. *niloticus*), and rainbow trout (*O*. *mykiss*) gut microbiota [18, 85–86]. Similarly, an enrichment in *Lactobacillus* genus was found in the intestine of trout fed with a diet containing insect meal, which is rich in chitin, a mucopolysaccharide polymer, which acts as a prebiotic being hardly digested by many fish species [48]. The combined administration of GMOS and phytogenic (GMOSPHYTO) also caused an enrichment in the *Bacteroides* and *Prevotella* genera in mucosa-associated microbial communities of sea bass. In humans, *Bacteroides* is clinically important genus, whereas the *Prevotella* species are commensal colonizers at mucosal sites, generally characterized by low pathogenicity and only few strains belonging to this genus exhibit opportunistic properties by promoting inflammatory diseases [87]. Interestingly, in line with our results, the number of counts of *Prevotella* was greater among mice fed galactomannan derived from citrus and fenugreek than among normal diet fed animals [88]. In the same study the increased amount of *Prevotella* was associated to an improved glucose metabolism.

The aforementioned differences in bacterial communities were also confirmed by the unweighted and weighted UniFrac analyses. PCoA plots showed that the major effect of diet was observed in faecal samples in terms of both, relative abundance and presence of specific taxa.

## Conclusions

In summary, CTRL feed-associated bacteria showed a higher diversity in comparison to the bacteria found in functional feeds. We hypothesize that the additives GMOS and PHYTO, having an antimicrobial effect, have favoured the dominance of specific taxa, such as LAB thus lowering the biodiversity of functional feeds. However, the high amount of Lactobacillus found in functional feeds is in terms of relative abundance and it is not an absolute quantification. It means that we cannot discriminate whether an enrichment in certain species is due to an increase in absolute abundance or a decrease in the abundances of other dominant taxa.

As for the gut-associated microbiota, our findings suggest that the dietary inclusion of GMOS (0.5%) and a phytogenic composed by garlic and labiatae plants oils (0.02%) in a low FM and FO diet induces changes in bacterial community composition of European sea bass. However, if on allochthonous microbiota the combined inclusion of GMOS and PHYTO showed an antagonistic effect, at mucosa level only GMOSPHYTO diet increased the relative abundance of the Bacteroidales, Lactobacillales and Clostridiales resident microbiota orders. The main beneficial effects of GMOS and PHYTO on gut microbiota are on one side, the reduction of coliforms and Vibrionales bacteria, which include several potentially pathogenic species for fish, and the other, the enrichment of gut microbiota composition with butyrate producer taxa. Therefore, it is evident that these functional ingredients have a great potential to be used as health-promoting agents in in the farming of European sea bass and other marine fish.

## Supporting information

S1 Dataset List of OTUs found in two macro-groups of samples. (XLSX)

S1 Fig Alpha diversity rarefaction curves of observed OTUs index of faeces and feed samples. (TIFF)

S2 Fig Alpha diversity rarefaction curves of observed OTUs index of mucosa and feed samples. (TIF)

S1 Table Original number of reads assigned to OTUs and alpha diversity metrics values (rarefied at 4500 reads) of feed-associated microbial community. Reported data are expressed as means ± SD (n = 4). The means were compared by Kruskal-Wallis test (p<0.05). Different superscript letters on the same column indicate significant differences.
(DOCX)

S2 Table Mean relative abundance (%) ± SD of the most prevalent phyla, orders, classes, families, and genera found in feed samples. Means in the same row with different letters indicate statistical significance between taxonomic groups’ abundances (p<0.05).
(DOCX)

## References

[1] Shepherd CJ, Jackson AJ. Global fishmeal and fish-oil supply: inputs, outputs and markets. J. Fish. Biol. 2013; 83:1046–1066. 10.1111/jfb.12224 24090562

[2] FAO. World Fisheries and Aquaculture, 2018.

[3] Francis G, Makkar HPS, Becker K. Anti-nutritional factors present in plant derived alternate fish feed ingredients and their effects in fish. Aquaculture 2001; 199:197–227. 10.1016/S0044-8486(01)00526-9

[4] Krogdahl Å, Penn M, Thorsen J, Refstie S, Bakke AM. Important antinutrients in plant feedstuffs for aquaculture: an update on recent findings regarding responses in salmonids. Aquac. Res. 2010; 41:333–344. 10.1111/j.1365-2109.2009.02426.x

[5] Øverland M, Sørensen M, Storebakken T, Penn M, Krogdahl Å, Skrede A. Pea protein concentrate substituting fish meal or soybean meal in diets for Atlantic salmon (Salmo salar). Effect on growth performance, nutrient digestibility, carcass composition, gut health, and physical feed quality. Aquaculture 2009; 288:305–311. 10.1016/j.aquaculture.2008.12.012

[6] Torrecillas S, Monpel D, Caballero MJ, Montero D, Merrifield D, Rodies A, et al Effect of fishmeal and fish oil replacement by vegetable meals and oils on gut health of European sea bass (Dicentrarchus labrax). Aquaculture 2017; 468:386–398. 10.1016/j.aquaculture.2016.11.005

[7] Benedito-Palos L, Navarro JC, Sitjà-Bobadilla A, Bell JG, Kaushik S, Pérez-Sánchez J. High levels of vegetable oils in plant protein-rich diets fed to gilthead sea bream (Sparus aurata L.): growth performance, muscle fatty acid profiles and histological alterations of target tissues. Br. J. Nutr. 2008; 100:992–1003. 10.1017/S0007114508966071 18377678

[8] Ringø E, Myklebust R, Mayhew TM, Olsen RE. Bacterial translocation and pathogenesis in the digestive tract of larvae and fry. Aquaculture 2007; 268:251–264. 10.1016/j.aquaculture.2007.04.047

[9] Torrecillas S, Montero D, Izquierdo M. Improved health and growth of fish fed mannan oligosaccharides: Potential mode of action. Fish Shellfish Immunol. 2014; 36:525–544. 10.1016/j.fsi.2013.12.029 24412165

[10] Song SK, Beck BR, Kim D, Park J, Kim J, Kim HD, et al Prebiotics as immunostimulants in aquaculture: A review. Fish Shellfish Immunol. 2014; 40:40–48. 10.1016/j.fsi.2014.06.016 24973515

[11] Rimoldi S, Finzi G, Ceccotti C, Girardello R, Grimaldi A, Ascione C, et al Butyrate and taurine exert a mitigating effect on the inflamed distal intestine of European sea bass fed with a high percentage of soybean meal. Fish. Aquat. Sci. 2016; 19:40 10.1186/s41240-016-0041-9

[12] Al-Sagheer AA, Mahmoud HK, Reda FM, Mahgoub SA, Ayyat MS. Supplementation of diets for Oreochromis niloticus with essential oil extracts from lemongrass (Cymbopogon citratus) and geranium (Pelargonium graveolens) and effects on growth, intestinal microbiota, antioxidant and immune activities. Aquacult. Nutr. 2018; 24:1006–1014. 10.1111/anu.12637

[13] Stevanović ZD, Bošnjak-Neumüller J, Pajić-Lijaković I, Raj J, Vasiljević M. Essential oils as feed additives-future perspectives. Molecules 2018; 23:1717 10.3390/molecules23071717 30011894 PMC6100314

[14] Encarnação P. Functional feed additives in aquaculture feeds In: Aquafeed formulation (ed. by Nates S. F.) Elsevier, Amsterdam 2016; 217–236. 10.1016/B978-0-12-800873-7.00005-1

[15] Grisdale-Helland B, Helland SJ, Gatlin DM. The effects of dietary supplementation with mannanoligosaccharide, fructooligosaccharide or galactooligosaccharide on the growth and feed utilization of Atlantic salmon (Salmo salar). Aquaculture 2008; 283:163–167. 10.1016/j.aquaculture.2008.07.012

[16] Ringø E, Olsen RE, Gifstad TØ, Dalmo RA, Amlund H, Hemre GI, et al Prebiotics in aquaculture: a review. Aquacult. Nutr. 2010; 16:117–36. 10.1111/j.1365-2095.2009.00731.x

[17] Zhou Q-C, Buentello JA, Gatlin DM. Effects of dietary prebiotics on growth performance, immune response and intestinal morphology of red drum (Sciaenops ocellatus). Aquaculture 2010; 309:253–257. 10.1016/j.aquaculture.2010.09.003

[18] Dimitroglou A, Merrifield DL, Spring P, Sweetman J, Moate R, Davies SJ. Effects of mannan oligosaccharide (MOS) supplementation on growth performance, feed utilisation, intestinal histology and gut microbiota of gilthead sea bream (Sparus aurata). Aquaculture 2010; 300(1): 182–188. 10.1016/j.aquaculture.2010.01.015

[19] Guerreiro I, Serra CR, Pousão-Ferreira P, Oliva-Teles A, Enes P. Prebiotics effect on growth performance, hepatic intermediary metabolism, gut microbiota and digestive enzymes of white sea bream (Diplodus sargus). Aquacult. Nutr. 2018a; 24:153–163. 10.1111/anu.12543

[20] Torrecillas S, Terova G, Makol A, Serradell A, Valdenegro V, Gini E, et al Dietary phytogenics and galactomannan oligosaccharides in low fish meal and fish oil-based diets for European sea bass (Dicentrarchus labrax) juveniles: Effects on gut health and implications on in vivo gut bacterial translocation. 2019; PlosOne. 14(9): e0222063 10.1371/journal.pone.0222063 31532807 PMC6750610

[21] Gibson GR, Roberfroid MB. Dietary modulation of the human colonic microflora introducing the concept of prebiotics. J. Nutr. 1995; 125:1401–1412. 10.1093/jn/125.6.1401 7782892

[22] Torrecillas S, Makol A, Caballero MJ, Montero D, Robaina L, Real F, et al Immune stimulation and improved infection resistance in European sea bass (Dicentrarchus labrax) fed mannan oligosaccharides. Fish Shellfish Immunol. 2007; 23:969–981. 10.1016/j.fsi.2007.03.007 17766145

[23] Torrecillas S, Makol A, Caballero MJ, Montero D, Ginés R, Sweetman J, et al Improved feed utilization, intestinal mucus production and immune parameters in sea bass (Dicentrarchus labrax) fed mannan oligosaccharides (MOS). Aquacult. Nutr. 2011; 17:223–233. 10.1111/j.1365-2095.2009.00730.x

[24] Torrecillas S, Makol A, Betancor MB, Montero D, Caballero MJ, Sweetman J, et al Enhanced intestinal epithelial barrier health status on European sea bass (Dicentrarchus labrax) fed mannan oligosaccharides. Fish Shellfish Immunol. 2013; 34:1485–1495. 10.1016/j.fsi.2013.03.351 23528875

[25] Torrecillas S, Montero D, Caballero MJ, Robaina L, Zamorano MJ, Sweetman J, et al Effects of dietary concentrated mannan oligosaccharides supplementation on growth, gut mucosal immune system and liver lipid metabolism of European sea bass (Dicentrarchus labrax) juveniles. Fish Shellfish Immunol. 2015a; 42:508–516. 10.1016/j.fsi.2014.11.033 25447638

[26] Torrecillas S, Montero D, Caballero MJ, Pittman KA, Custódio M, Campo A, et al Dietary mannan oligosaccharides: counteracting the side effects of soybean meal oil inclusion on European sea bass (Dicentrarchus labrax) gut health and skin mucosa mucus production? Front. Immunol. 2015b; 6:397 10.3389/fimmu.2015.00397 26300883 PMC4525062

[27] Torrecillas S, Caballero MJ, Montero D, Sweetman J, Izquierdo M. Combined effects of dietary mannan oligosaccharides and total fish oil substitution by soybean oil on European sea bass (Dicentrarchus labrax) juvenile diets. Aquacult. Nutr. 2016; 22:1079–1090. 10.1111/anu.12322

[28] Torrecillas S, Rivero-Ramírez F, Izquierdo MS, Caballero MJ, Makol A, Suarez-Bregua P, et al Feeding European sea bass (Dicentrarchus labrax) juveniles with a functional synbiotic additive (mannan oligosaccharides and Pediococcus acidilactici): An effective tool to reduce low fishmeal and fish oil gut health effects? Fish Shellfish Immunol. 2018; 81:10–20. 10.1016/j.fsi.2018.07.007 29981880

[29] Terova G, Forchino A, Rimoldi S, Brambilla F, Antonini M, Saroglia M. Bio-Mos®: An effective inducer of dicentracin gene expression in European sea bass (Dicentrarchus labrax). Comp. Biochem. Physiol. 2009; Part B, 153:372–377. 10.1016/j.cbpb.2009.04.008 19393760

[30] Dimitroglou A, Merrifield DL, Carnevali O, Picchietti S, Avella M, Daniels C, et al Microbial manipulations to improve fish health and production–A Mediterranean perspective. Fish Shellfish Immunol. 2011; 30:1–16. 10.1016/j.fsi.2010.08.009 20801223

[31] Guerreiro I, Oliva-Teles A, Enes P. Prebiotics as functional ingredients: focus on Mediterranean fish aquaculture. Rev. Aquac. 2018b; 10:800–832. 10.1111/raq.12201

[32] Butt RL, Volkoff H. Gut microbiota and energy homeostasis in fish. Front. Endocrinol. 2019; (Lausanne) 10 10.3389/fendo.2019.00009 30733706 PMC6353785 30733706

[33] Wong JM, de Souza R, Kendall CW, Emam A, Jenkins DJ. Colonic health: fermentation and short chain fatty acids. J. Clin. Gastroenterol. 2006; 40(3):235–43. 10.1097/00004836-200603000-00015 16633129

[34] Roberfroid M, Gibson GR, Hoyles L, McCartney AL, Rastall R, Rowland I, et al Prebiotic effects: metabolic and health benefits. Br. J. Nutr. 2010; 104:S1–S63. 10.1017/S0007114510003363 20920376

[35] Zeng Z, Zhang S, Wang H, Piao X. Essential oil and aromatic plants as feed additives in non-ruminant nutrition: A review. J. Anim. Sci. Biotechnol. 2015; 6(1):7 10.1186/s40104-015-0004-5 25774291 PMC4359495

[36] Harikrishnan R, Balasundaram C, Heo M. Impact of plant products on innate and adaptive immune system of cultured finfish and shellfish. Aquaculture 2011; 317:1–15. 10.1016/j.aquaculture.2011.03.039

[37] Chakraborty SB, Horn P, Hancz C. Application of phytochemicals as growth-promoters and endocrine modulators in fish culture. Rev. Aquacult. 2014; 6:1–19. 10.1111/raq.12021

[38] Reverter M, Bontemps N, Lecchini D, Banaigs B, Sasal P. Use of plant extracts in fish aquaculture as an alternative to chemotherapy: current status and future perspectives. Aquaculture 2014; 433:50–61. 10.1016/j.aquaculture.2014.05.048

[39] Zaki MA, Labib EM, Nour AM, Tonsy HD, Mahmoud SH. Effect of some medicinal plants diets on mono sex Nile tilapia (Oreochromis niloticus), growth performance, feed utilization and physiological parameters. APCBEE Procedia 2012; 4:220–227. 10.1016/j.apcbee.2012.11.037

[40] Awad E, Cerezuela R, Esteban MÁ. Effects of fenugreek (Trigonella foenum graecum) on gilthead seabream (Sparus aurata L.) immune status and growth performance. Fish Shellfish Immunol. 2015; 45:454–464. 10.1016/j.fsi.2015.04.035 25956720

[41] Sutili FJ, de Lima Silva L, Gressler LT, Gressler LT, Battisti EK, Heinzmann BM, et al Plant essential oils against Aeromonas hydrophila: *in vitro* activity and their use in experimentally infected fish. J. Appl. Microbiol. 2015; 119(1):47–54. 10.1111/jam.12812 25810355

[42] Sutili FJ, Gatlin DM, Heinzmann BM, Baldisserotto B. Plant essential oils as fish diet additives: benefits on fish health and stability in feed. Rev. Aquac. 2018; 10:716–726. 10.1111/raq.12197

[43] da Cunha JA, Heinzmann BM, Baldisserotto B. The effects of essential oils and their major compounds on fish bacterial pathogens—a review. J. Appl. Microbiol. 2018; 125:328–344. 10.1111/jam.13911 29742307

[44] Hüsnü K, Baser C, Demirci F. Chemistry of essential oils In: Flavours and fragrances: chemistry, bioprocessing and sustainability. Edited by Berger RG. New York: Springer 2007; 43–86. 10.1007/978-3-540-49339-6_4

[45] Burt SA. Essential oils: their antibacterial properties and potential applications in foods–a review. Int. J. Food Microbiol. 2004; 94:223–253. 10.1016/j.ijfoodmicro.2004.03.022 15246235

[46] Giannenas I, Triantafillou E, Stavrakakis S, Margaroni M, Mavridis S, Steiner T, et al Assessment of dietary supplementation with carvacrol or thymol containing feed additives on performance, intestinal microbiota and antioxidant status of rainbow trout (Oncorhynchus mykiss). Aquaculture 2012; 350-353-26–32. 10.1016/j.aquaculture.2012.04.027

[47] Rimoldi S, Gini E, Iannini F, Gasco L, Terova G. The effects of dietary insect meal from Hermetia illucens prepupae on autochthonous gut microbiota of rainbow trout (Oncorhynchus mykiss). Animals 2019; 9(4):143 10.3390/ani9040143 30987067 PMC6523354

[48] Terova G, Rimoldi S, Ascione C, Gini E, Ceccotti C, Gasco L. Rainbow trout (Oncorhynchus mykiss) gut microbiota is modulated by insect meal from Hermetia illucens prepupae in the diet. Rev. Fish Biol. Fisheries 2019; 29:465–486. 10.1007/s11160-019-09558-y

[49] Takahashi S, Tomita J, Nishioka K, Hisada T, Nishijima M. Development of a prokaryotic universal primer for simultaneous analysis of bacteria and archaea using next-generation sequencing. 2014; PLoS ONE 9(8), e105592 10.1371/journal.pone.0105592 25144201 PMC4140814

[50] Bolyen E, Rideout JR, Dillon MR, Bokulich NA, Abnet C, Al-Ghalith GA, et al QIIME 2: Reproducible, interactive, scalable, and extensible microbiome data science. 2018; PeerJ Preprints 6, e27295v2 10.7287/peerj.preprints.27295v2 PMC7015180 31341288

[51] Callahan BJ, McMurdie PJ, Rosen MJ, Han AW, Johnson AJA, Holmes SP. DADA2: High-resolution sample inference from Illumina amplicon data. 2016; Nat. Methods 13:581–583. 10.1038/nmeth.3869 27214047 PMC4927377

[52] Lozupone C, Knight R. UniFrac: a New Phylogenetic Method for Comparing Microbial Communities. Appl. Environ. Microbiol. 2005; 71:8228–8235. 10.1128/AEM.71.12.8228-8235.2005 16332807 PMC1317376

[53] Lozupone CA, Hamady M, Kelley ST, Knight R. Quantitative and qualitative diversity measures lead to different insights into factors that structure microbial communities. Appl. Environ. Microbiol. 2007; 73:1576–1585. 10.1128/AEM.01996-06 17220268 PMC1828774

[54] Hammer DAT, Ryan PD, Hammer Ø, Harper DAT. Past: Paleontological Statistics Software Package for Education and Data Analysis. Palaeontol. Electron. 2001; 4(1): 4–9. http://palaeo-electronica.org

[55] Dawood MAO, Koshio S, Esteban MA. Beneficial roles of feed additives as immunostimulants in aquaculture: a review. Rev. Aquacult. 2018; 10: 950–974. 10.1111/raq.12209

[56] Carda-Diéguez M, Mira A, Fouz B. Pyrosequencing survey of intestinal microbiota diversity in cultured sea bass (Dicentrarchus labrax) fed functional diets. FEMS Microbiol. Ecol. 2014; 87:451–459. 10.1111/1574-6941.12236 24730648

[57] Kim D-H, Brunt J, Austin B. Microbial diversity of intestinal contents and mucus in rainbow trout (Oncorhynchus mykiss). J. Appl. Microbiol. 2007; 102:1654–1664. 10.1111/j.1365-2672.2006.03185.x 17578431

[58] Wu S, Gao T, Zheng Y, Wang W, Cheng Y, Wang G. Microbial diversity of intestinal contents and mucus in yellow catfish (Pelteobagrus fulvidraco). Aquaculture 2010; 303:1–7. 10.1016/j.aquaculture.2009.12.025

[59] Rimoldi S, Terova G, Ascione C, Giannico R, Brambilla F. Next generation sequencing for gut microbiome characterization in rainbow trout (Oncorhynchus mykiss) fed animal by-product meals as an alternative to fishmeal protein sources. 2018; PLoS ONE 13(3):e0193652 10.1371/journal.pone.0193652 29509788 PMC5839548

[60] Gatesoupe F-J, Huelvan C, Le Bayon N, Le Delliou H, Madec L, Mouchel O, et al The highly variable microbiota associate to intestinal mucosa correlates with growth and hypoxia resistance of sea bass, *Dicentrarchus labrax*, submitted to different nutritional histories. BMC Microbiology 2016; 16:266 10.1186/s12866-016-0885-2 27821062 PMC5100225

[61] Etyemez M, Balcázarb JL, Demirkalea I, Dikela S. Effects of garlic-supplemented diet on growth performance and intestinal microbiota of rainbow trout (Oncorhynchus mykiss). Aquaculture 2018; 486: 170–174. 10.1016/j.aquaculture.2017.12.022

[62] Sekirov I, Russell SL, Caetano L, Antunes M, Finlay BB. Gut microbiota in health and disease. Physiol. Rev. 2010; 90: 859–904. 10.1152/physrev.00045.2009 20664075

[63] Apper E, Weissman D, Respondek F, Guyonvarch A, Baron F, Boisot P, et al Hydrolysed wheat gluten as part of a diet based on animal and plant proteins supports good growth performance of Asian seabass (Lates calcarifer), without impairing intestinal morphology or microbiota. Aquaculture 2016; 453: 40–48. 10.1016/j.aquaculture.2015.11.018

[64] Dimitroglou A, Merrifield DL, Moate R, Davies SJ, Spring P, Sweetman J, et al Dietary mannan oligosaccharide supplementation modulates intestinal microbial ecology and improves gut morphology of rainbow trout, Oncorhynchus mykiss (Walbaum). J. Anim. Sci. 2009; 87: 3226–3234. 10.2527/jas.2008-1428 19617514

[65] Ran C, Hu J, Liu W, Liu Z, He S, Dan BC, et al Thymol and carvacrol affect hybrid tilapia through the combination of direct stimulation and an intestinal microbiota-mediated effect: insights from a germ-free zebrafish model. J. Nutr. 2016; 146(5): 1132–40. 10.3945/jn.115.229377 27075912

[66] Guardiola FA, Bahi A, Bakhrouf A, Esteban MA. Effects of dietary supplementation with fenugreek seeds, alone or in combination with probiotics, on gilthead seabream (Sparus aurata L.) skin mucosal immunity. Fish Shellfish Immunol. 2017; 65: 169–178. 10.1016/j.fsi.2017.04.014 28433714

[67] Torrecillas S, Makol A, Caballero MJ, Montero D, Dhanasiri AKS, Sweetman J, et al Effect of mortality and stress response in European sea bass, Dicentrarchus labrax (L.), fed mannan oligosaccharides (MOS) after Vibrio anguillarum exposure. J. Fish Dis. 2012; 35: 591–602. 10.1111/j.1365-2761.2012.01384.x 22690841

[68] Spring P, Wenk C, Dawson KA, Newman KE. The effects of dietary mannan oligosaccharides on cecal parameters and the concentrations of enteric bacteria in the cecae of Salmonella-challenged broiler chicks. Poult. Sci. 2000; 79:205–211. 10.1093/ps/79.2.205 10735748

[69] Ringø E, Strøm E, Tabachek JA. Intestinal microflora of salmonids: a review. Aquac. Nutr. 1995; 26: 773–789. 10.1111/j.1365-2109.1995.tb00870.x

[70] Pryde SE, Duncan SH, Hold GL, Stewart CS, Harry JF. The microbiology of butyrate formation in the human colon. FEMS Microbiol. Lett. 2002; 217: 133–139. 10.1111/j.1574-6968.2002.tb11467.x 12480096

[71] Esquivel-Elizondo S, Ilhan ZE, Garcia-Penã EI, Krajmalnik-Brown R. Insights into butyrate production in a controlled fermentation system via gene predictions. 2017; mSystems 2: e00051–17. 10.1128/mSystems.00051-17 28761933 PMC5516221

[72] Walker AW, Ince J, Duncan SH, Webster LM, Holtrop G, Ze X, et al Dominant and diet-responsive groups of bacteria within the human colonic microbiota. ISME J. 2011; 5(2): 220–30. 10.1038/ismej.2010.118 20686513 PMC3105703

[73] Ze X, Duncan SH, Louis P, Flint HJ. Ruminococcus bromii is a keystone species for the degradation of resistant starch in the human colon. ISME J. 2012; 6(8): 1535–43. 10.1038/ismej.2012.4 22343308 PMC3400402

[74] Bonder MJ, Tigchelaa EF, Cai X, Trynka G, Cenit MC, Hrdlickova B, et al The influence of a short-term gluten-free diet on the human gut microbiome. Genome Med. 2016; 8: 45 10.1186/s13073-016-0295-y 27102333 PMC4841035

[75] Ferreira-Halder CV, de Sousa Faria AV, Andrade SS. Action and function of Faecalibacterium prausnitzii in health and disease. Best Pract. Res. Cl. Ga. 2017; 31: 643–648. 10.1016/j.bpg.2017.09.011 29566907

[76] Cho JH, Kim HJ, Kim IH. Effects of phytogenic feed additive on growth performance, digestibility, blood metabolites, intestinal microbiota, meat color and relative organ weight after oral challenge with Clostridium perfringens in broilers. Livest. Sci. 2014; 160: 82–88. 10.1016/j.livsci.2013.11.006

[77] Mountzouris KC, Paraskevas V, Tsirtsikos P, Palamidi I, Steiner T, Schatzmayr G, et al Assessment of a phytogenic feed additive effect on broiler growth performance, nutrient digestibility and caecal microflora composition. Anim. Feed Sci. Tech. 2011; 168: 223–231. 10.1016/j.anifeedsci.2011.03.020

[78] Franciosini MP, Casagrande-Proietti P, Forte C, Beghelli D, Acuti G, Zanichelli D, et al Effects of oregano (Origanum vulgare L.) and rosemary (Rosmarinus officinalis L.) aqueous extracts on broiler performance, immune function and intestinal microbial population. J. Appl. Anim. Res. 2016; 44(1): 474–479. 10.1080/09712119.2015.1091322

[79] Giannenas I, Bonos E, Christaki E, Florou-Paneri P. Essential oils and their applications in animal nutrition. Med. Aromat. Plants. 2013; 2: 1–12. 10.4172/2167-0412.1000140

[80] Helander IM, Alakomi HL, Latva-Kala K, Mattıla-Sandholm T, Pol I, Smıd EJ, et al Characterization of the action of selected essential oil components on gram-negative bacteria. J. Agric. Food Chem. 1998; 46: 3590–3595. 10.1021/jf980154m

[81] Ankri S, Mirelman D. Antimicrobial properties of allicin from garlic. Microbes Infect. 1999; 1(2): 125–129. 10.1016/s1286-4579(99)80003-3 10594976

[82] Nazzaro F, Fratianni F, De Martino L, Coppola R, De Feo V. Effect of essential oils on pathogenic bacteria. Pharmaceuticals (Basel) 2013; 6: 1451–74. 10.3390/ph6121451 24287491 PMC3873673

[83] Ringø E, Gatesoupe F-J. Lactic acid bacteria in fish: a review. Aquaculture 1998; 160: 177–203. 10.1016/S0044-8486(97)00299-8

[84] Kim SK, Bhatnagar I, Kang KH. Development of marine probiotics: prospects and approach. Adv. Food Nutr. Res. 2012; 65: 353–362. 10.1016/B978-0-12-416003-3.00023-8 22361199

[85] Gonçalves AT, Gallardo-Escárate C. Microbiome dynamic modulation through functional diets based on pre- and probiotics (mannan-oligosaccharides and Saccharomyces cerevisiae) in juvenile rainbow trout (Oncorhynchus mykiss). J. Appl. Microbiol. 2017; 122: 1333–1347. 10.1111/jam.13437 28256031

[86] Levy-Pereira N, Yasui GS, Vedovelli Cardozo M, Neto JD, Vaz Farias TH, Sakabe R, et al Immunostimulation and increase of intestinal lactic acid bacteria with dietary mannan-oligosaccharide in Nile tilapia juveniles. R. Bras. Zootec. 2018; 47: e20170006 10.1590/rbz4720170006

[87] Larsen JM. The immune response to Prevotella bacteria in chronic inflammatory disease. Immunology. 2017; 151(4): 363–374. 10.1111/imm.12760 28542929 PMC5506432

[88] Shtriker MG, Hahn M, Taieb E, Nyska A, Moallem U, Tirosh O, et al Fenugreek galactomannan and citrus pectin improve several parameters associated with glucose metabolism and modulate gut microbiota in mice. Nutrition. 2018; 46: 134–142. 10.1016/j.nut.2017.07.012 28993009

