# Supplementary figures and images for: Assessment of dietary supplementation with galactomannan oligosaccharides and phytogenics on gut microbiota of European sea bass (Dicentrarchus Labrax) fed low fishmeal and fish oil based diet
Source: PLoS One. 2020 Apr 16;15(4):e0231494. doi: 10.1371/journal.pone.0231494 (PMC7162502; doi:10.1371/journal.pone.0231494)

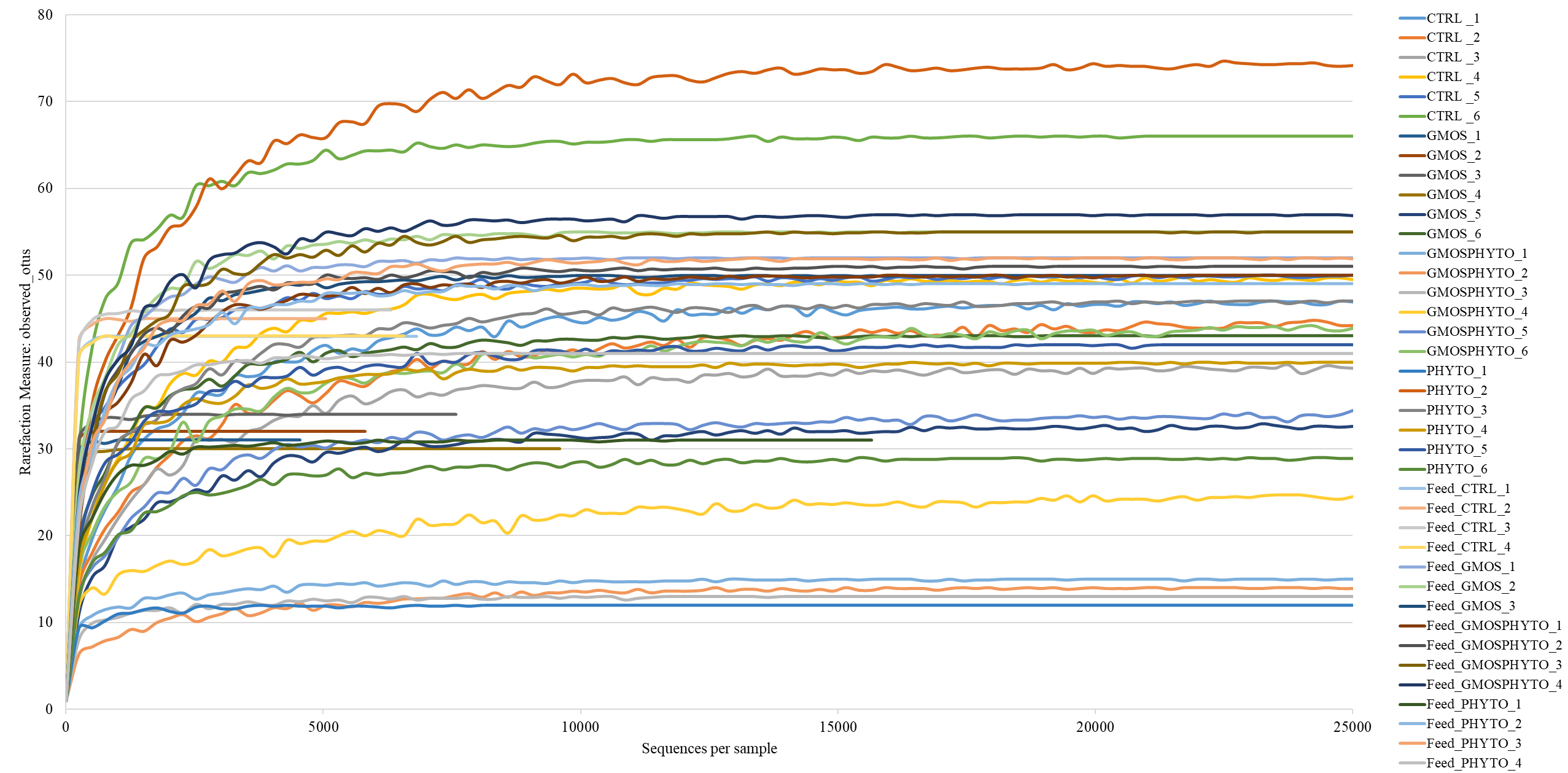

Supplement: S1 Fig — (TIFF) [file pone.0231494.s002.tiff]

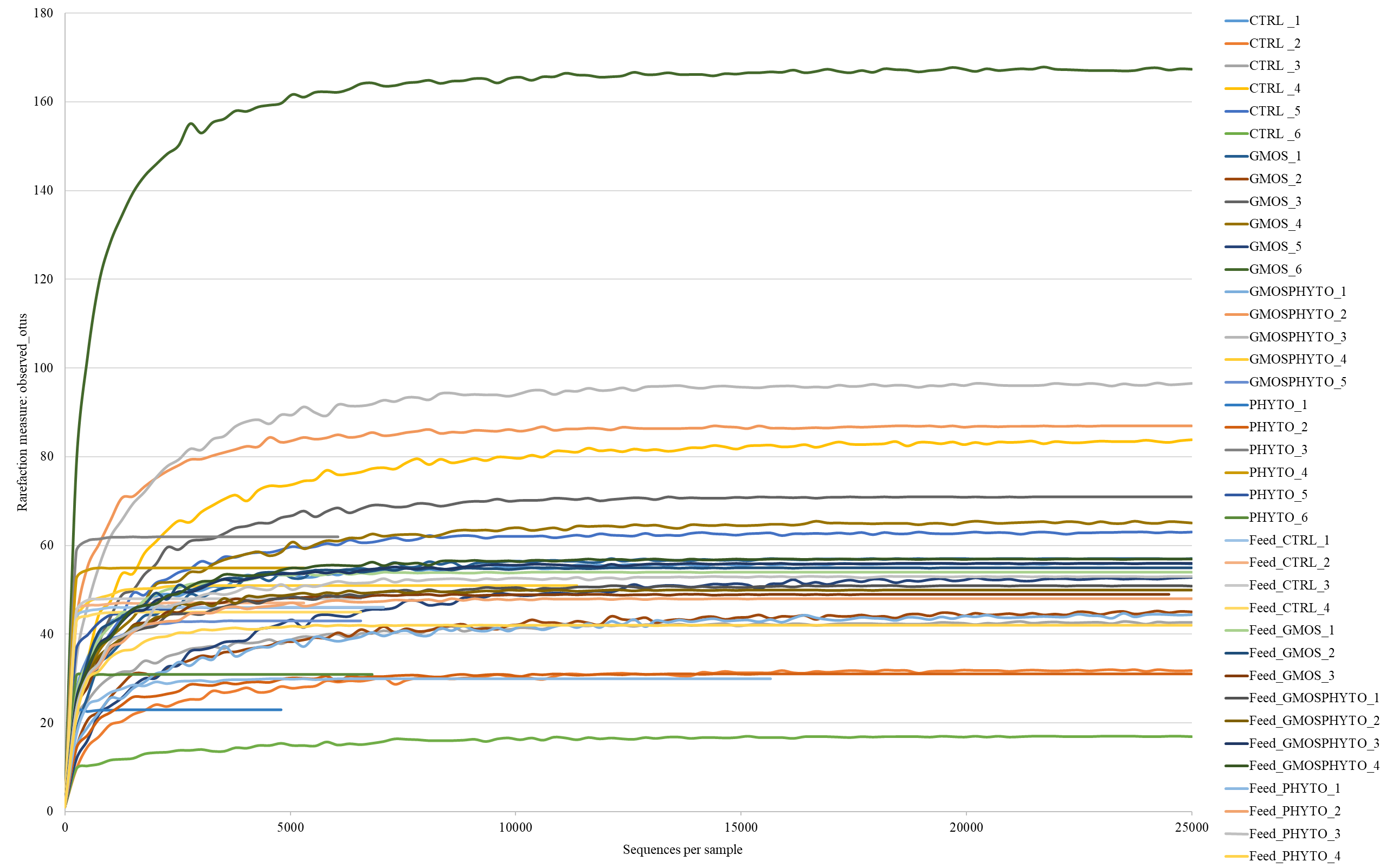

Supplement: S2 Fig — (TIF) [file pone.0231494.s003.tif]
