# Supplementary material for: Assessment of dietary supplementation with galactomannan oligosaccharides and phytogenics on gut microbiota of European sea bass (Dicentrarchus Labrax) fed low fishmeal and fish oil based diet
Source: PLoS One. 2020 Apr 16;15(4):e0231494. doi: 10.1371/journal.pone.0231494 (PMC7162502; doi:10.1371/journal.pone.0231494)
Supplement: S1 Table — Reported data are expressed as means ± SD (n = 4). The means were compared by Kruskal-Wallis test (p<0.05). Different superscript letters on the same column indicate significant differences. (DOCX) [file pone.0231494.s004.docx]

**S1 Table**. Original number of reads assigned to OTUs and alpha diversity metrics values (rarefied at 4500 reads) of feed-associated microbial community. Reported data are expressed as means ± SD (n = 4). The means were compared by Kruskal-Wallis test (p < 0.05). Different superscript letters on the same column indicate significant differences.

|  | **FEEDS** | | | |
| --- | --- | --- | --- | --- |
| **Item** | CTRL | GMOS | GMOSPHYTO | PHYTO |
| Reads | 6,315 ± 750^b^ | 30,613 ± 5,521^a^ | 42,689 ± 8558^a^ | 30,953 ± 11,762^a^ |
| Observed OTUs | 44.25 ± 1.50 | 50.67 ± 2.08 | 50.00 ± 1.83 | 42.00 ± 8.41 |
| Shannon | 5.18 ± 0.07^a^ | 2.87 ± 0.10^b^ | 2.81 ± 0.05^b^ | 2.67 ± 0.16^b^ |
| Pielou’s evenness | 0.95 ± 0.00^a^ | 0.50 ± 0.01^b^ | 0.50 ± 0.00^b^ | 0.50 ± 0.01^b^ |
| Faith PD | 3.57 ± 1.50 | 4.14 ± 0.28 | 4.06 ± 0.14 | 3.71 ± 0.46 |
