# Supplementary material for: Assessment of dietary supplementation with galactomannan oligosaccharides and phytogenics on gut microbiota of European sea bass (Dicentrarchus Labrax) fed low fishmeal and fish oil based diet
Source: PLoS One. 2020 Apr 16;15(4):e0231494. doi: 10.1371/journal.pone.0231494 (PMC7162502; doi:10.1371/journal.pone.0231494)
Supplement: S2 Table — Means in the same row with different letters indicate statistical significance between taxonomic groups’ abundances (p<0.05). (DOCX) [file pone.0231494.s005.docx]

**S2 Table.** Mean relative abundance (%) ± SD of the most prevalent phyla, orders, classes, families, and genera found in feed samples. Means in the same row with different letters indicate statistical significance between taxonomic groups’ abundances (p<0.05).

| **TAXA** |  |  |  |  |  |  | **FEEDS** | | |  |  |  |  |  |  |
| --- | --- | --- | --- | --- | --- | --- | --- | --- | --- | --- | --- | --- | --- | --- | --- |
|  | CTRL | | |  | GMOS | | |  | GMOSPHYTO | | |  | PHYTO | | |
| **Phylum** |  |  |  |  |  |  |  |  |  |  |  |  |  |  |  |
| *Actinobacteria* | 3.45 | ± | 0.87^a^ |  | 0.25 | ± | 0.01^ab^ |  | 0.19 | ± | 0.02^b^ |  | 0.17 | ± | 0.09^b^ |
| *Firmicutes* | 49.06 | ± | 3.68^b^ |  | 54.94 | ± | 4.30^ab^ |  | 60.19 | ± | 2.30^a^ |  | 61 | ± | 3.72^a^ |
| *Fusobacteria* | 7.16 | ± | 1.55^b^ |  | 10.79 | ± | 1.10^a^ |  | 8.98 | ± | 1.00^ab^ |  | 9.27 | ± | 1.31^ab^ |
| *Proteobacteria* | 40.28 | ± | 4.23^a^ |  | 33.94 | ± | 3.40^ab^ |  | 30.57 | ± | 1.30^ab^ |  | 29.5 | ± | 2.35^b^ |
|  |  |  |  |  |  |  |  |  |  |  |  |  |  |  |  |
| **Class** |  |  |  |  |  |  |  |  |  |  |  |  |  |  |  |
| *Actinobacteria* | 3.45 | ± | 0.87^a^ |  | 0.25 | ± | 0.01^ab^ |  | 0.19 | ± | 0.02^b^ |  | 0.17 | ± | 0.09^b^ |
| *Bacilli* | 36.77 | ± | 5.13^b^ |  | 51.48 | ± | 4.63^a^ |  | 57.13 | ± | 2.55^a^ |  | 58 | ± | 3.90^a^ |
| *Clostridia* | 12.28 | ± | 2.14^a^ |  | 3.15 | ± | 0.23^ab^ |  | 2.77 | ± | 0.25^b^ |  | 2.74 | ± | 0.29^b^ |
| *Fusobacteriia* | 7.16 | ± | 1.55^b^ |  | 10.79 | ± | 1.10^a^ |  | 8.98 | ± | 1.00^ab^ |  | 9.27 | ± | 1.31^ab^ |
| *Alphaproteobacteria* | 9.95 | ± | 1.46^b^ |  | 27.75 | ± | 2.89^a^ |  | 25.45 | ± | 1.07^a^ |  | 24.4 | ± | 1.76^ab^ |
| *Betaproteobacteria* | 7.18 | ± | 1.08^a^ |  | 0.08 | ± | 0.09^ab^ |  | 0.01 | ± | 0.02^b^ |  | 0.01 | ± | 0.01^b^ |
| *Gammaproteobacteria* | 23.15 | ± | 3.75^a^ |  | 6.11 | ± | 0.49^ab^ |  | 5.1 | ± | 0.38^b^ |  | 5.07 | ± | 0.65^b^ |
|  |  |  |  |  |  |  |  |  |  |  |  |  |  |  |  |
| **Order** |  |  |  |  |  |  |  |  |  |  |  |  |  |  |  |
| *Actinomycetales* | 3.45 | ± | 0.87^a^ |  | 0.25 | ± | 0.01^ab^ |  | 0.19 | ± | 0.02^b^ |  | 0.17 | ± | 0.09^b^ |
| *Bacillales* | 6.45 | ± | 1.75^a^ |  | 0.95 | ± | 0.07^ab^ |  | 0.65 | ± | 0.09^b^ |  | 1.1 | ± | 0.20^a^ |
| *Lactobacillales* | 30.32 | ± | 3.54^b^ |  | 50.53 | ± | 4.58^ab^ |  | 56.47 | ± | 2.58^a^ |  | 56.9 | ± | 4.09^a^ |
| *Clostridiales* | 12.28 | ± | 2.14^a^ |  | 3.15 | ± | 0.23^ab^ |  | 2.77 | ± | 0.25^b^ |  | 2.74 | ± | 0.29^b^ |
| *Fusobacteriales* | 7.16 | ± | 1.55^b^ |  | 10.79 | ± | 1.10^a^ |  | 8.98 | ± | 1.00^ab^ |  | 9.27 | ± | 1.31^ab^ |
| *Rickettsiales* | 9.95 | ± | 1.46^b^ |  | 27.65 | ± | 2.88^a^ |  | 25.36 | ± | 1.08^a^ |  | 24.4 | ± | 1.74^ab^ |
| *Burkholderiales* | 7.18 | ± | 1.08^a^ |  | 0.04 | ± | 0.08^b^ |  | 0 | ± | 0.00^b^ |  | 0 | ± | 0.00^b^ |
| *Alteromonadales* | 8.92 | ± | 1.90^a^ |  | 2.29 | ± | 0.20^ab^ |  | 2.07 | ± | 0.15^b^ |  | 2.02 | ± | 0.33^b^ |
| *Enterobacteriales* | 4.15 | ± | 0.53^a^ |  | 0.43 | ± | 0.19^b^ |  | 0.46 | ± | 0.12^b^ |  | 0.4 | ± | 0.06^b^ |
| *Vibrionales* | 10.08 | ± | 3.69^a^ |  | 3.17 | ± | 0.14^ab^ |  | 2.42 | ± | 0.17^b^ |  | 2.58 | ± | 0.29^b^ |
|  |  |  |  |  |  |  |  |  |  |  |  |  |  |  |  |
| **Family** |  |  |  |  |  |  |  |  |  |  |  |  |  |  |  |
| *Corynebacteriaceae* | 3.84 | ± | 1.02^a^ |  | 0 | ± | 0.00^b^ |  | 0.01 | ± | 0.03^b^ |  | 0.02 | ± | 0.04^b^ |
| *Bacillaceae* | 4.29 | ± | 3.24 |  | 1.28 | ± | 0.05 |  | 0.87 | ± | 0.13 |  | 1.44 | ± | 0.28 |
| *Planococcaceae* | 2.87 | ± | 2.03^a^ |  | 0.04 | ± | 0.06^b^ |  | 0 | ± | 0.00^b^ |  | 0.02 | ± | 0.04^b^ |
| *Enterococcaceae* | 5.55 | ± | 0.48^a^ |  | 0.22 | ± | 0.08^ab^ |  | 0.01 | ± | 0.03^b^ |  | 0.10 | ± | 0.15^b^ |
| *Lactobacillaceae* | 17.76 | ± | 4.52^b^ |  | 66.18 | ± | 3.95^ab^ |  | 73.37 | ± | 2.70^a^ |  | 72.8 | ± | 3.86^a^ |
| *Leuconostocaceae* | 6.82 | ± | 1.43^a^ |  | 1.74 | ± | 0.36^ab^ |  | 1.23 | ± | 0.07^b^ |  | 1.13 | ± | 0.18^b^ |
| *Clostridiaceae* | 6.55 | ± | 0.90^a^ |  | 2.07 | ± | 0.30^ab^ |  | 1.68 | ± | 0.15^b^ |  | 1.73 | ± | 0.31^b^ |
| *Lachnospiraceae* | 0.00 | ± | 0.00 |  | 0.58 | ± | 0.03 |  | 0.53 | ± | 0.06 |  | 0.47 | ± | 0.18 |
| *Peptostreptococcaceae* | 7.10 | ± | 1.62 |  | 0.00 | ± | 0.00 |  | 0.00 | ± | 0.00 |  | 0.00 | ± | 0.00 |
| *Fusobacteriaceae* | 7.96 | ± | 1.72^b^ |  | 14.95 | ± | 1.97^a^ |  | 12.05 | ± | 1.49^a^ |  | 12.3 | ± | 1.97^a^ |
| *Comamonadaceae* | 7.96 | ± | 1.09 |  | 0.06 | ± | 0.11 |  | 0.00 | ± | 0.00 |  | 0.00 | ± | 0.00 |
| *Shewanellaceae* | 9.91 | ± | 2.13^a^ |  | 3.18 | ± | 0.39^ab^ |  | 2.77 | ± | 0.21^b^ |  | 2.68 | ± | 0.49^b^ |
| *Enterobacteriaceae* | 4.61 | ± | 0.59^a^ |  | 0.60 | ± | 0.28^b^ |  | 0.62 | ± | 0.17^b^ |  | 0.53 | ± | 0.07^b^ |
| *Vibrionaceae* | 11.2 | ± | 4.09^a^ |  | 4.39 | ± | 0.36^ab^ |  | 3.25 | ± | 0.27^b^ |  | 3.42 | ± | 0.45^b^ |
|  |  |  |  |  |  |  |  |  |  |  |  |  |  |  |  |
| **Genus** |  |  |  |  |  |  |  |  |  |  |  |  |  |  |  |
| *Corynebacterium* | 3.84 | ± | 1.02 |  | 0.00 | ± | 0.00 |  | 0.01 | ± | 0.03 |  | 0.02 | ± | 0.04 |
| *Bacillus* | 4.29 | ± | 3.24 |  | 0.91 | ± | 0.12 |  | 0.59 | ± | 0.09 |  | 1.31 | ± | 0.29 |
| *Rummeliibacillus* | 2.87 | ± | 2.03 |  | 0.04 | ± | 0.06 |  | 0.00 | ± | 0.00 |  | 0.02 | ± | 0.04 |
| *Enterococcus* | 5.55 | ± | 0.48^a^ |  | 0.22 | ± | 0.08^ab^ |  | 0.01 | ± | 0.03^b^ |  | 0.10 | ± | 0.15^b^ |
| *Lactobacillus* | 17.76 | ± | 4.52^b^ |  | 66.18 | ± | 3.95^ab^ |  | 73.37 | ± | 2.70^a^ |  | 72.8 | ± | 3.86^a^ |
| *Cetobacterium* | 5.24 | ± | 0.92^a^ |  | 2.82 | ± | 0.34^ab^ |  | 1.99 | ± | 0.31^b^ |  | 2.18 | ± | 0.37^b^ |
| *Fusobacterium* | 2.71 | ± | 2.09^b^ |  | 12.06 | ± | 1.70^a^ |  | 10.00 | ± | 1.18^a^ |  | 10.10 | ± | 1.65^a^ |
| *Comamonas* | 7.96 | ± | 1.09 |  | 0.06 | ± | 0.11 |  | 0.00 | ± | 0.00 |  | 0.00 | ± | 0.00 |
| *Shewanella* | 9.91 | ± | 2.13^a^ |  | 3.18 | ± | 0.39^ab^ |  | 2.77 | ± | 0.21^b^ |  | 2.68 | ± | 0.49^b^ |
| *Erwinia* | 4.61 | ± | 0.59^a^ |  | 0.60 | ± | 0.28^b^ |  | 0.59 | ± | 0.14^b^ |  | 0.53 | ± | 0.07^b^ |
| *Photobacterium* | 11.2 | ± | 4.09^a^ |  | 4.02 | ± | 0.34^ab^ |  | 3.25 | ± | 0.27^b^ |  | 3.24 | ± | 0.41^b^ |
